# HIV/AIDS knowledge and stigmatizing attitudes among adult residents of the United Arab Emirates: A cross-sectional study

**DOI:** 10.1371/journal.pone.0362376

**Published:** 2026-11-06

**Authors:** Hiba Jawdat Barqawi, Kamel A. Samara, Abdul Rahman Sheiko, Mohamed Alhariri, Moustafa Saber Mashal, Amal Abu Helwa, Eman Abu-Gharbieh

**Affiliations:** 1 Department of Clinical Sciences, College of Medicine, University of Sharjah, Sharjah, United Arab Emirates; 2 Research Institute of Medical and Health Sciences, University of Sharjah, Sharjah, United Arab Emirates; 3 Department of Internal Medicine, Jefferson Abington Hospital, Abington, United States of America; 4 School of Pharmacy, The University of Jordan, Amman, Jordan; University of Technology Sydney, AUSTRALIA

## Abstract

**Background:**

HIV-related stigma remains a significant barrier to prevention and treatment efforts in the Middle East and North Africa (MENA) region. This study assessed HIV knowledge and stigmatizing attitudes among United Arab Emirates (UAE) residents.

**Methods:**

A cross-sectional survey was conducted across the UAE between January and March 2025. A total of 1,211 eligible adult residents were recruited through convenience sampling. Demographic analyses included all 1,211 participants, while HIV knowledge and stigma analyses included 1,171 participants with analyzable outcome data. The questionnaire assessed demographics, HIV transmission knowledge, and stigmatizing attitudes using the Stigmatizing Attitudes Towards People Living with HIV/AIDS Scale (SAT-PLWHA-S). Data were analyzed using descriptive statistics, bivariate analyses, and multiple linear regression.

**Results:**

Among the 1,211 participants included in the study, 1,171 contributed analyzable data to the HIV knowledge and stigma analyses. While 96.4% had heard of HIV/AIDS and demonstrated moderately high transmission knowledge (mean score: 75.5%), significant misconceptions persisted, particularly regarding condom effectiveness (60.5% overestimated risk). Strong stigmatizing attitudes emerged, especially regarding criminalization of transmission (79.6% support) and moralistic views. Higher knowledge correlated with less stigma (r = 0.347, p < 0.001), though nationality, language preference, and knowing someone with HIV were stronger predictors. Only 6.2% knew someone with HIV.

**Conclusions:**

Despite adequate basic knowledge, significant HIV-related stigma persists in the UAE, driven by cultural and moral perspectives. Comprehensive interventions combining education, contact-based strategies, and policy reform are needed to reduce stigma and improve HIV prevention outcomes.

## Introduction

HIV remains a major global public health challenge. In 2025, an estimated 40.9 million people worldwide were living with HIV. Despite advances in treatment and prevention, HIV remains a public health concern due to continued transmission and the social stigma associated with the disease [1]. The Middle East and North Africa region has a comparatively low overall HIV prevalence, with an estimated prevalence of 0.07% [0.06–0.10%] among adults aged 15–49 years in 2024. However, contrary to global trends showing declining rates of new infections, the MENA region is experiencing a concerning increase in HIV cases [2,3]. Between 2001 and 2012, the region saw a 73% increase in newly diagnosed individuals, the highest rate of increase globally [4]. Since 2010, new infections have increased by 10–21% in the region, making it one of the few areas worldwide experiencing this alarming trend [5]. HIV infection in this area is largely concentrated among key populations, including people who inject drugs (PWID), men who have sex with men (MSM), and female sex workers (FSWs) [2,6]. However, comprehensive data collection and surveillance remain significant challenges due to social, cultural, and religious taboos surrounding HIV/AIDS and the behaviors associated with its transmission. Under-reporting is widespread, and obtaining exact statistics is difficult as HIV remains a controversial topic in many MENA countries [7].

Studies in the Arabian Peninsula have documented varying levels of HIV/AIDS knowledge, with one systematic review reporting an overall knowledge level of 74.4% [1]. Although basic awareness of HIV/AIDS is generally high in the region, misconceptions about its transmission remain common. These include beliefs that HIV can spread through mosquito bites, sharing meals, or casual contact with an infected person [6]. HIV-related stigma in the MENA region is particularly pronounced due to the interplay between religion, culture, and geopolitics [4]. HIV infection may be the most stigmatized medical condition in the Arab world [8]. Although some studies have reported improvements in attitudes over time, the reasons for these changes remain unclear [6]. Stigma has profound implications for HIV prevention and treatment, acting as a barrier to testing, disclosure, and care-seeking behaviors.

Critical gaps exist in research on HIV knowledge and stigma in the MENA region. The region is often described as a “missing piece” in the global HIV/AIDS map in terms of epidemiological data [2]. Access to HIV diagnosis and treatment in the MENA region remains substantially below global targets. In 2024, an estimated 63% of people living with HIV in the region knew their HIV status, 48% were receiving antiretroviral therapy, and 44% had achieved viral suppression. Research on stigma and its determinants is particularly limited in the United Arab Emirates (UAE), despite its importance for developing effective interventions. This study aims to address these gaps by assessing HIV knowledge and stigmatizing attitudes among residents of the UAE. Specifically, the study seeks to: (1) evaluate the level of knowledge regarding HIV transmission and prevention; (2) assess the presence and dimensions of HIV-related stigma using the Stigmatizing Attitudes Towards People Living with HIV/AIDS Scale (SAT-PLWHA-S); (3) examine the relationship between HIV knowledge and stigmatizing attitudes; and (4) identify sociodemographic predictors of HIV knowledge and stigmatizing attitudes. By addressing these objectives, this study will contribute to the limited body of research on HIV knowledge and stigma in the UAE and provide evidence to inform targeted interventions for reducing HIV-related stigma and improving HIV prevention efforts in the region.

## Methodology

### Study design

This cross-sectional study was conducted across the UAE between 2^nd^ January and 31^st^ March 2025. Participants were recruited through convenience sampling using WhatsApp, Instagram, and X. Current UAE residency was assessed through a mandatory self-report screening question at the beginning of the questionnaire. Only respondents who confirmed that they currently lived in the UAE were eligible for inclusion. A minimum sample size was calculated using Cochran’s formula: $n=\frac{z^{\text{2}}p(\text{1}-p)}{E^{\text{2}}}$, with the sampling error taken to be 5% and p = 0.5 for maximum. n represents the needed sample size, while p is the expected prevalence, which was taken to be 0.5, given no previous studies have covered this topic in the country previously, all yielding a minimum sample size of 385.

Participants were presented with a detailed information sheet outlining the study objectives and procedures before participation. They needed to confirm electronically that they had read the information, agreed to participate, and were at least 18 years old. No identifying information was collected to ensure anonymity. The study was conducted in accordance with the approved protocol and applicable ethical standards. Electronic informed consent was obtained from all participants before they accessed the questionnaire. No directly identifying information was collected, and access to the study data was restricted to the research team. This study was reviewed and approved by the Research Ethics Committee at the University of Sharjah (Reference Number: REC-24-02-14-01-F). It was conducted in accordance with all relevant guidelines and regulations.

Eligible participants were individuals aged 18 years or older who currently resided in the UAE and provided electronic informed consent. Both UAE nationals and expatriate residents were eligible. Respondents who did not confirm that they were at least 18 years old, did not currently reside in the UAE, or did not provide electronic informed consent were not eligible to participate. For outcome-specific analyses, participants without analyzable responses for the relevant HIV knowledge or stigma variables were excluded from that analysis.

### Questionnaire development

The questionnaire used in this study was developed by adapting existing validated instruments and adding specific items relevant to the UAE context. The instrument comprised four main sections. 1. Demographics: Age, sex, education level, marital status, nationality, place of residence, field of work, and health insurance status. 2. HIV Knowledge and Prior Familiarity: Previous awareness of HIV/AIDS, self-assessed knowledge level, and personal acquaintance with someone living with HIV/AIDS. 3. HIV Transmission Knowledge: A seven-item scale assessing perceived risk of HIV transmission in various scenarios, including casual contact and sexual contact (with and without condom use). Responses were measured on a four-point scale from “very low risk” to “very high risk.” 4. HIV-related Stigma: The Stigmatizing Attitudes Towards People Living with HIV/AIDS Scale (SAT-PLWHA-S) developed by Beaulieu et al. (2014) [9] was adapted for this study. The scale consists of 27 items across seven dimensions: concerns about occasional encounters, avoidance of personal contact, responsibility and blame, liberalism, non-discrimination, confidentiality of serological status, and criminalization of HIV transmission. Responses were measured on a four-point Likert scale from “strongly disagree” to “strongly agree.” The questionnaire was developed in English and Arabic to accommodate the multilingual population of the UAE. Forward and backward translation was conducted to ensure conceptual equivalence between the two versions. Both language versions were pilot tested with 20 volunteers as a pragmatic pretest to assess item clarity, cultural appropriateness, conceptual equivalence between the Arabic and English versions, questionnaire flow, and approximate completion time. The pilot was intended to identify wording, translation, and usability problems rather than to provide formal psychometric validation. Minor wording and formatting revisions were made based on participant feedback. Both questionnaires can be found in the supplementary material S1 File.

### Statistical analysis

Data were exported to CSV format and analyzed using Python (version 3.9) with pandas, NumPy, Matplotlib, seaborn, and statsmodels packages. Descriptive statistics were calculated for all variables, including frequencies and percentages for categorical data and means with standard deviations for continuous data. HIV transmission knowledge was scored on a scale of 1–4 for each item, with higher scores indicating more accurate knowledge. HIV transmission knowledge was assessed using seven transmission-risk scenarios. Responses to each item were scored on a four-point scale, with higher scores representing responses closer to the prespecified correct risk category for that scenario. Item scores were averaged to generate a composite HIV transmission knowledge score, which was converted to a 0–100 scale. Higher scores indicated greater HIV transmission knowledge. Knowledge was analyzed as a continuous variable, and no cutoff was used to classify participants as having “good,” “poor,” “adequate,” or “inadequate” knowledge. The SAT-PLWHA-S items were scored on a four-point Likert scale. For positively worded, non-stigmatizing statements, responses were coded from 1 for “strongly disagree” to 4 for “strongly agree.” Stigmatizing or negatively worded items were reverse-coded so that higher values consistently represented less stigmatizing attitudes. Mean scores were calculated for each theoretical stigma dimension and for the overall scale. The overall stigma score was analyzed as a continuous variable; no cutoff was used to classify participants as having positive or negative attitudes. Therefore, a higher mean score indicated less HIV-related stigma, whereas a lower score indicated greater HIV-related stigma. Bivariate analyses included t-tests and ANOVA to compare knowledge and stigma scores across different demographic groups. Pearson’s correlation coefficient was calculated to examine the relationship between HIV knowledge and stigmatizing attitudes. Multiple linear regression was conducted to identify predictors of stigmatizing attitudes, with the total stigma score as the dependent variable and knowledge score, demographic characteristics, and other relevant variables as independent predictors. The regression model was exploratory, intended to identify candidate predictors rather than to test a pre-specified confirmatory model. Formal assessment of regression assumptions (multicollinearity, normality of residuals, homoscedasticity) was not performed; results should be interpreted with this limitation in mind. Statistical significance was set at p < 0.05 for all analyses. Missing data were handled through pairwise deletion, with the number of valid responses reported for each analysis.

## Results

### Demographics

A total of 1,211 eligible participants were included in the demographic analyses. Of these, 1,171 had complete analyzable responses for the HIV knowledge and stigma outcomes. Forty participants were excluded from those analyses because their outcome data were incomplete. Of 1,211 participants, 60.2% (n = 729/1,211) were female, 62.8% (n = 761/1,211) were young adults aged 18–25 years, and 53.8% (n = 652/1,211) had a diploma or bachelor’s degree. Nearly three-quarters of participants were single (73.7%, n = 892/1,211). Regarding nationality, 46.9% (n = 568/1,211) identified as other Arab (non-UAE), 30.4% (n = 368/1,211) as UAE nationals, and 22.7% (n = 275/1,211) as non-Arab. More than half of participants (53.8%, n = 651/1,211) resided in Sharjah and other northern emirates. In terms of occupation, 25.5% (n = 309/1,211) were students in non-health related fields, 17.8% (n = 216/1,211) were health sciences students, and 7.5% (n = 91/1,211) were healthcare professionals. The majority of participants (78.0%, n = 944/1,211) reported having health insurance. The survey was completed in English by 62.7% (n = 759/1,211) of participants and in Arabic by 37.3% (n = 452/1,211). Table 1 presents the complete demographic characteristics of study participants.

**Table 1:** Demographics and HIV awareness characteristics of participants.

Characteristic	% (n)	Characteristic	% (n)
**Sex**		**Field of work**	
Female	60.2% (n=729)	Healthcare professionals	7.5% (n=91)
Male	39.8% (n=482)	Non-healthcare professionals	34.4% (n=417)
**Age group**		Housewife	6.1% (n=74)
Young adult (18-25 years)	62.8% (n=761)	Students (healthcare)	17.8% (n=216)
Adult (26-44 years)	29.3% (n=355)	Students (non-healthcare)	25.5% (n=309)
Middle adult or older (≥45 years)	7.8% (n=95)	Unemployed	8.6% (n=104)
**Education level**		**Have you heard about AIDS before?**	
High school or lower	34.1% (n=413)	Yes	96.4% (n=1,167)
Diploma/Bachelor’s degree	53.8% (n=652)	No	3.6% (n=44)
Postgraduate degree	12.1% (n=146)	**How knowledgeable are you about AIDS?**	
**Marital status**		Not at all/slightly	27.3% (n=330)
Single	73.7% (n=892)	Moderately	47.0% (n=569)
Married/ Others	26.3% (n=319)	Very/extremely	25.8% (n=312)
**Nationality**		**Do you know anyone with AIDS?**	
UAE national	30.4% (n=368)	Yes	6.2% (n=75)
Other Arab	46.9% (n=568)	No	93.8% (n=1,136)
Non-Arab	22.7% (n=275)	**Can a healthy-looking person have AIDS?**	
**Place of residence**		Yes	79.0% (n=925/1,171)
Abu Dhabi	20.6% (n=250)	No	5.2% (n=61/1,171)
Dubai	25.6% (n=310)	I don’t know	15.8% (n=185/1,171)
Sharjah and other northern emirates	53.8% (n=651)	**Language of survey**	
**Health insurance**		English	62.7% (n=759)
Yes	78.0% (n=944)	Arabic	37.3% (n=452)
No	22.0% (n=267)		

Sample size N = 1,211, except for variables for which a different denominator is specifically indicated

### HIV knowledge and prior familiarity

The vast majority of participants (96.4%, n = 1,167/1,211) reported having heard about HIV/AIDS previously. When asked about their self-assessed knowledge level, 47.0% (n = 569/1,211) reported being moderately knowledgeable, 27.3% (n = 330/1,211) reported being not at all or slightly knowledgeable, and 25.8% (n = 312/1,211) reported being very or extremely knowledgeable about HIV/AIDS. Only 6.2% (n = 75/1,211) of participants reported knowing someone with HIV/AIDS. Regarding whether a healthy-looking person can have HIV, 79.0% (n = 925/1,171) correctly answered yes, while 15.8% (n = 185/1,171) reported they did not know.

Assessment of HIV transmission knowledge revealed varying levels of accuracy across different scenarios (Table 2). Participants demonstrated high accuracy in understanding the risk of transmission through unprotected sexual intercourse, with 88.7% (n = 1,039/1,171) correctly identifying this as a “very high” risk. Similarly, good knowledge was observed for understanding the low risk of casual contact, with 66.5% (n = 779/1,171) correctly identifying “very low” risk for shaking hands and 61.1% (n = 716/1,171) for working in the same office. However, significant misconceptions were evident. For sharing a glass with an infected person, 28.9% (n = 338/1,171) incorrectly assessed this as high or very high risk when the actual risk is very low. Most concerning, 60.5% (n = 708/1,171) of participants overestimated the risk of protected sex with condom use as high or very high, when the correct assessment is low risk. The overall mean HIV knowledge score was 75.5% (SD = 19.3), indicating a moderately high level of knowledge about HIV transmission risks.

**Table 2:** HIV Transmission Knowledge: Perceived Risk by Transmission Scenario (N = 1,171). * denotes the correct level of risk for each scenario.

Transmission scenario	Very low % (n)	Low % (n)	High % (n)	Very high % (n)
**Casual contact scenarios**				
Kissing on the cheek	60.0% (n = 703)*	28.2% (n = 330)	8.4% (n = 98)	3.4% (n = 40)
Shaking hands	66.5% (n = 779)*	25.5% (n = 299)	5.8% (n = 68)	2.1% (n = 25)
Working in same office	61.1% (n = 716)*	28.4% (n = 333)	8.2% (n = 96)	2.2% (n = 26)
**Fluid contact scenarios**				
Sharing a glass	42.5% (n = 498)*	28.6% (n = 335)	19.2% (n = 225)	9.7% (n = 113)
Being coughed/sneezed on	40.6% (n = 475)*	30.0% (n = 351)	19.1% (n = 224)	10.3% (n = 121)
**Sexual contact scenarios**				
Sex with condom	6.8% (n = 79)	32.8% (n = 384)*	46.2% (n = 541)	14.3% (n = 167)
Sex without condom	2.1% (n = 25)	1.8% (n = 21)	7.3% (n = 86)	88.7% (n = 1,039)*
**Knowledge Summary**				
Mean overall knowledge score (0–100 scale)			75.5% (SD = 19.3)	
Correctly identified very low risk for kissing			60.0% (n = 703/1,171)	
Correctly identified very high risk for unprotected sex			88.7% (n = 1,039/1,171)	
Overestimated risk for sharing a glass (high/very high)			28.9% (n = 338/1,171)	
Overestimated risk for protected sex (high/very high)			60.5% (n = 708/1,171)	

### HIV-related stigma attitudes

Mean scores across the seven theoretical stigma dimensions revealed varying levels of stigmatizing attitudes (Table 3). The dimension with the lowest score, indicating highest stigma, was criminalization of transmission (M = 1.89, SD = 0.78), followed by liberalism (M = 2.01, SD = 0.83). The highest scores, indicating lowest stigma, were observed for responsibility and blame (M = 2.73, SD = 0.71) and non-discrimination (M = 2.55, SD = 0.71). The overall mean stigma score was 2.32 (SD = 0.45). Fig 1 displays the distribution of mean scores across all seven stigma dimensions using a radar chart, illustrating the areas of highest and lowest stigma while Fig 2 shows the distribution of total stigma scores across the sample.

**Table 3:** HIV stigma attitudes: Response distribution for key items by dimension (N = 1,171). *Scores coded so that higher values (1-4 scale) indicate less stigmatizing attitudes (less stigma).

Stigma item	Strongly disagree % (n)	Disagree % (n)	Agree % (n)	Strongly agree % (n)
**Concerns about occasional encounters**				
*Being around someone with AIDS does NOT bother me*	17.5% (n=205)	31.3% (n=367)	34.0% (n=398)	17.2% (n=201)
*NOT worried for health if coworker had AIDS*	23.0% (n=269)	30.9% (n=362)	29.7% (n=348)	16.4% (n=192)
*NOT bother if AIDS clinic on my street*	8.0% (n=94)	17.3% (n=203)	39.2% (n=459)	35.4% (n=415)
**Avoidance of personal contact**				
*I could NOT be friends with someone with AIDS*	28.7% (n=336)	38.3% (n=449)	21.4% (n=251)	11.5% (n=135)
*I would limit contact with infected person*	18.9% (n=221)	29.1% (n=341)	34.2% (n=401)	17.8% (n=208)
*I would NOT hug someone with AIDS*	20.7% (n=242)	30.5% (n=357)	28.4% (n=333)	20.4% (n=239)
**Responsibility and blame**				
*Drug users deserve to have AIDS*	46.0% (n=539)	33.9% (n=397)	13.0% (n=152)	7.1% (n=83)
*Support depends on how person was infected*	25.4% (n=297)	26.2% (n=307)	31.7% (n=371)	16.7% (n=196)
*Disgusted by homosexual transmission*	18.3% (n=214)	18.3% (n=214)	21.0% (n=246)	42.4% (n=497)
*People without condom deserve what they get*	28.4% (n=333)	41.6% (n=487)	17.0% (n=199)	13.0% (n=152)
*People with AIDS only themselves to blame*	27.9% (n=327)	44.0% (n=515)	18.5% (n=216)	9.7% (n=113)
*Most people with AIDS are responsible*	19.7% (n=231)	40.7% (n=476)	28.4% (n=332)	11.3% (n=132)
**Liberalism**				
*Young people must not have sex to fight AIDS*	14.9% (n=175)	24.4% (n=286)	27.5% (n=322)	33.1% (n=388)
*Traditional sexual values help control AIDS*	9.6% (n=112)	12.2% (n=143)	39.6% (n=464)	38.6% (n=452)
*AIDS emergence linked to sexual freedom*	8.5% (n=100)	11.5% (n=135)	34.2% (n=400)	45.8% (n=536)
*AIDS spread linked to moral decline*	11.8% (n=138)	19.6% (n=229)	33.0% (n=386)	35.7% (n=418)
**Non-discrimination**				
*Right to work serving public*	28.5% (n=334)	27.4% (n=321)	28.7% (n=336)	15.4% (n=180)
*Infected children should go to daycare*	17.6% (n=206)	24.6% (n=288)	35.2% (n=412)	22.6% (n=265)
*Doctors with AIDS should work with patients*	26.1% (n=305)	31.0% (n=363)	30.3% (n=355)	12.6% (n=148)
*Should NOT be allowed to live/work in UAE*	29.5% (n=345)	40.6% (n=475)	17.5% (n=205)	12.5% (n=146)
*NOT bother if roommate was infected*	28.5% (n=334)	38.3% (n=448)	23.1% (n=270)	10.2% (n=119)
**Confidentiality of status**				
*Right to know if someone around me infected*	5.2% (n=61)	15.2% (n=178)	36.1% (n=423)	43.5% (n=509)
*Test results should remain confidential*	12.1% (n=142)	25.2% (n=295)	40.9% (n=479)	21.8% (n=255)
*Doctors should report names to government*	11.4% (n=133)	19.0% (n=222)	34.0% (n=398)	35.7% (n=418)
**Criminalization of transmission**				
*Transmission should be punishable by law*	11.5% (n=135)	22.6% (n=265)	33.8% (n=396/1171)	32.0% (n=375/1171)
*Knowing transmission makes them criminals*	6.7% (n=78)	12.4% (n=145)	21.9% (n=256/1171)	59.1% (n=692/1171)
*Transmitting AIDS is a crime*	6.2% (n=72)	18.2% (n=213)	31.7% (n=371/1171)	44.0% (n=515/1171)

Sample size N = 1,171. Percentages are based on the original response categories. When calculating dimension and overall scores, items were coded so that higher scores indicated less stigmatizing attitudes.

**Fig 1:**
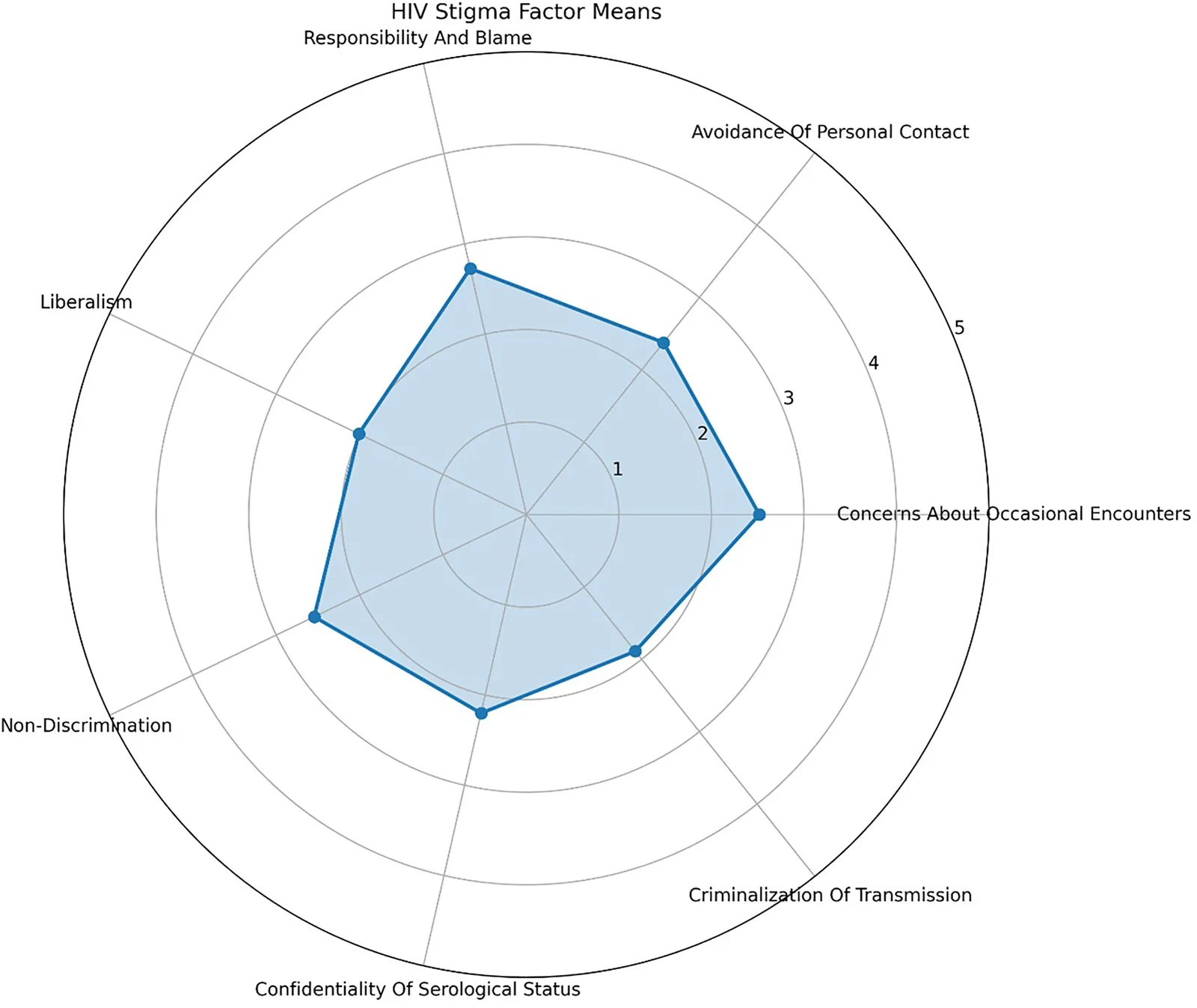
The average values across the seven dimensions of HIV stigma.

**Fig 2:**
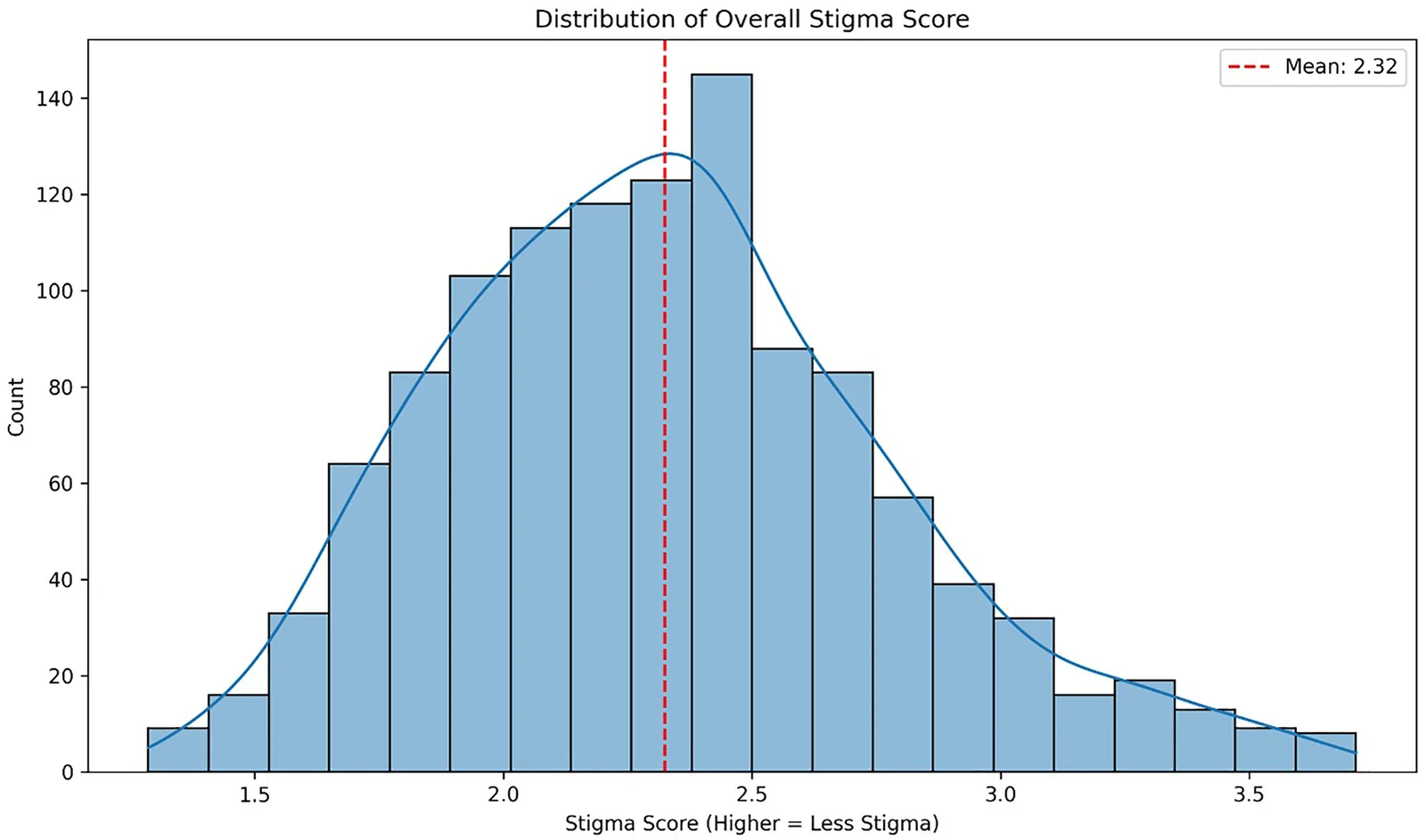
The average overall stigma score with higher values representing less stigmatizing views.

Examination of specific stigma items revealed concerning patterns of attitudes. Regarding criminalization, 79.6% (n = 948/1,171) agreed or strongly agreed that “people who know they are infected with AIDS and transmit the virus are criminals,” and 75.7% (n = 886/1,171) agreed or strongly agreed that “transmitting the AIDS virus is a crime.” High levels of moralistic attitudes were evident, with 79.9% (n = 936/1,171) agreeing that “the emergence of AIDS is associated with increased sexual freedom” and 78.2% (n = 916/1,171) agreeing that “reinforcement of traditional sexual values will help control AIDS.” Regarding personal contact with people living with HIV (PLWH), 52.2% (n = 611/1,171) reported discomfort at having a roommate who was HIV-positive, and 52.0% (n = 609/1,171) indicated they would limit their contact with someone they knew was infected with HIV. Additionally, 48.9% (n = 572/1,171) stated they would not hug someone with AIDS. However, some positive attitudes were also observed: 74.6% (n = 874/1,171) supported the notion that healthcare workers would NOT be bothered by a clinic for people with AIDS on their street, and 79.9% (n = 936/1,171) disagreed or strongly disagreed that people who inject drugs deserve to have AIDS.

Significant differences in stigma scores were observed across demographic groups. ANOVA revealed substantial differences by nationality (F = 85.77, p < 0.001), language preference (F = 188.87, p < 0.001), education level (F = 4.02, p = 0.018), and field of work (F = 4.69, p = 0.003). Healthcare professionals and health sciences students showed marginally less stigmatizing attitudes compared to non-healthcare participants, though this difference did not reach statistical significance (t = 1.67, p = 0.096).

### Relationship between HIV knowledge and stigma

A significant positive correlation was found between HIV knowledge scores and total stigma scores (r = 0.347, p < 0.001), indicating that higher knowledge about HIV transmission was associated with less stigmatizing attitudes. However, the strength of this correlation varied substantially across stigma dimensions. The strongest positive correlations were observed for concerns about occasional encounters (r = 0.443) and non-discrimination (r = 0.416), while a negative correlation was found with avoidance of personal contact (r = −0.378). The weakest correlation was observed for criminalization of transmission (r = 0.046), suggesting that attitudes toward criminalizing HIV transmission were largely independent of HIV transmission knowledge. Interestingly, participants who knew someone with HIV demonstrated significantly lower stigma scores compared to those who did not (t = −4.09, p < 0.001). However, no significant difference was found in HIV knowledge between these groups (t = −0.75, p = 0.453), suggesting that the stigma-reducing effect of knowing someone with HIV operates independently of knowledge about transmission.

Multiple linear regression analysis identified several significant predictors of stigmatizing attitudes (Table 4). The model explained 27.5% of the variance in total stigma scores (adjusted R^2^ = 0.268, F = 39.97, p < 0.001). HIV knowledge was the strongest predictor of less stigmatizing attitudes (β = 0.007, p < 0.001). Nationality was strongly associated with stigmatizing attitudes, with both UAE nationals (β = −0.231, p < 0.001) and other Arabs (β = −0.254, p < 0.001) showing more stigmatizing attitudes compared to non-Arab participants. Arabic language preference was also associated with more stigmatizing attitudes (β = −0.201, p < 0.001). Male participants demonstrated slightly more stigmatizing attitudes than females (β = −0.057, p = 0.016). Knowing someone with HIV was significantly associated with less stigmatizing attitudes (β = 0.139, p = 0.003). Self-assessed knowledge level, age, and education level were not significant predictors in the multivariate model.

**Table 4:** Multiple linear regression predicting total HIV stigma scores (N = 1,171). Significant results are bold. CI: Confidence Interval; SE: Standard Error.

HIV Stigma – Oridnary Least Squares Regression (OLS)						
Model terms		β-coefficient	SE	t-statistic	P-value	95% CI
Intercept (*β*_0_)		**2.094**	**0.065**	**32.324**	**<0.0005**	**[1.967, 2.221]**
HIV knowledge		**0.007**	**0.001**	**10.511**	**<0.0005**	**[0.005, 0.008]**
Sex	Female	-	-	-	-	-
	**Male**	**-0.057**	**0.023**	**-2.422**	**0.016**	**[-0.103, -0.011]**
Age group	Adults (26-44 years)	-	-	-	-	-
	Middle adult/older (≥45 years)	-0.063	0.046	-1.374	0.170	[-0.152, 0.027]
	Young adult (18-25 years)	0.054	0.029	1.894	0.059	[-0.002, 0.111]
Education level	Diploma/Bachelor’s degree	-	-	-	-	-
	High school or lower	-0.050	0.027	-1.874	0.061	[-0.102, 0.002]
	Postgraduate degree	0.035	0.037	0.950	0.342	[-0.038, 0.108]
Nationality	Non-Arab	-	-	-	-	-
	**Other Arab**	**-0.254**	**0.031**	**-8.132**	**<0.0005**	**[-0.315, -0.193]**
	**UAE national**	**-0.231**	**0.035**	**-6.655**	**<0.0005**	**[-0.299, -0.163]**
Knows someone with HIV	No	-	-	-	-	-
	**Yes**	**0.139**	**0.047**	**2.945**	**0.003**	**[0.046, 0.232]**
Survey language	English	-	-	-	-	-
	**Arabic**	**-0.201**	**0.027**	**-7.385**	**<0.0005**	**[-0.254, -0.147]**
R^2^: 27.5%	Adjusted R^2^: 26.8%		F (10, 1,160) = 39.97; P< 0.0005			

## Discussion

The study included 1,211 adult UAE residents from different demographic groups. However, because participants were recruited through convenience sampling using social media and the sample included a high proportion of young adults, the findings should not be interpreted as nationally representative of all UAE residents. While the vast majority (96.4%) had heard about HIV/AIDS and demonstrated moderately high knowledge about transmission (mean score: 75.5%), significant stigmatizing attitudes persisted across multiple dimensions. When examining HIV-related stigma, strong support emerged for criminalization of transmission, with nearly 80% viewing knowing transmission as criminal behavior, alongside pervasive moralistic views linking HIV to sexual freedom and moral decline. Notably, more than half of participants expressed discomfort with close personal contact with people living with HIV, such as having an HIV-positive roommate. Regarding information sources, participants reported varying levels of trust in healthcare providers, with nationality, language preference, and personal connection to someone with HIV emerging as significant predictors of stigmatizing attitudes.

Our multivariate analysis revealed important insights into the predictors of HIV-related stigma in the UAE. The finding that higher HIV knowledge was significantly associated with less stigmatizing attitudes (β = 0.007, p < 0.001) provides evidence that educational interventions may be effective in reducing certain forms of stigma. However, the relatively modest effect size suggests that knowledge alone is insufficient to address deeply rooted stigmatizing attitudes. The strong associations with nationality and language preference—with UAE nationals (β = −0.231, p < 0.001) and other Arabs (β = −0.254, p < 0.001) showing more stigmatizing attitudes compared to non-Arabs—reflect the cultural and religious context in which HIV/AIDS is understood in the region. These findings suggest that public health interventions must be culturally sensitive and address the underlying socio-cultural factors that contribute to stigma, rather than relying solely on biomedical education.

Our findings align with broader regional patterns documented across the Middle East and North Africa. Recent studies indicate that HIV/AIDS stigmatization is particularly pronounced in the MENA region due to the interplay between religion, culture, and geopolitics, with HIV potentially being the most stigmatized medical condition in the Arab world [4,8]. While basic knowledge about HIV/AIDS is generally established in MENA populations, comprehensive knowledge remains low and negative attitudes toward people living with HIV are widespread [1,6]. In the Arabian Peninsula specifically, a meta-analysis found that while overall HIV/AIDS knowledge was 74.4%, the percentage for positive attitudes was only 52.8%, with even medical doctors showing high knowledge (94.1%) but positive attitudes in only 32.5% of cases. This regional context helps explain why we observed the disconnect between relatively high knowledge scores and persistent stigmatizing attitudes in our UAE sample.

The concentration of HIV among key populations in the MENA region is also relevant to our findings. Some of the attitudes captured in our survey, particularly those concerning sexual behavior, drug use, and responsibility for infection, may reflect stigma toward these groups as well as stigma toward HIV itself. However, our study was not designed to separate these influences or examine the experiences of key populations directly. This is an area that warrants further investigation.

HIV/AIDS stigma is a critical barrier to achieving global HIV prevention and treatment goals. Stigma contributes to underreporting of cases, delays in testing and diagnosis, poor treatment adherence, and reduced quality of life for people living with HIV [10,11]. In the MENA region specifically, access to HIV testing, treatment, and care remains well below the global average, with only 67% of people living with HIV knowing their status and only 50% having access to treatment in 2022 [12]. The region is falling far short of the 95-95-95 targets set by UNAIDS, which aim for 95% of people living with HIV to know their status, 95% of those diagnosed to receive sustained antiretroviral therapy, and 95% of those on treatment to achieve viral suppression by 2025. Stigma and discrimination are recognized as major obstacles to achieving these targets, as they deter individuals from seeking testing and care and contribute to social isolation of those living with HIV [13].

The development and validation of stigma measurement tools has been identified as a research priority for understanding and addressing HIV stigma [14]. The SAT-PLWHA-S used in our study was specifically designed to capture multiple dimensions of stigma, including stereotypes, prejudices, and discrimination, as well as social distancing and support for coercive measures [9].

This study’s results illustrate how residents of the UAE had relatively good understanding of HIV transmission risks in some scenarios (such as unprotected sex and casual contact through handshaking) but had notable misconceptions regarding other transmission routes. The finding that 60.5% of participants overestimated the risk of protected sex is particularly concerning, as this misconception may undermine confidence in condoms as an effective HIV prevention method. Similar knowledge gaps have been reported in studies from other MENA countries, where misconceptions about HIV transmission remain prevalent despite general awareness of the disease [2,5]. This result is significant given that accurate knowledge about transmission routes is fundamental to both preventing new infections and reducing irrational fears that fuel stigma.

As such, any public health initiatives need to address the need for comprehensive education that goes beyond basic awareness to tackle specific misconceptions. The finding that only 6.2% of participants knew someone with HIV highlights the “hidden” nature of the epidemic in the UAE, likely driven by stigma and fear of disclosure. This lack of personal contact represents a missed opportunity for stigma reduction, as contact theory suggests that positive interactions with stigmatized groups can reduce prejudice [15]. In fact, our regression analysis confirmed this, with knowing someone with HIV significantly associated with less stigmatizing attitudes (β = 0.139, p = 0.003). Lack of awareness about the realities of living with HIV in the era of effective antiretroviral therapy was also prevalent in our sample. The strong support for criminalization of transmission (79.6% viewing knowing transmission as criminal) reflects punitive attitudes that may actually increase HIV transmission by discouraging testing and disclosure. Research on HIV criminalization globally has shown that such laws do not reduce HIV transmission and may in fact be counterproductive to public health goals [16]. These findings mirror patterns observed in other conservative settings where HIV is viewed through a moral lens rather than solely as a health issue.

The differential impact of knowledge on various stigma dimensions revealed in our correlation analysis is particularly noteworthy. While knowledge showed strong positive correlations with reduced concerns about occasional encounters (r = 0.443) and support for non-discrimination (r = 0.416), it showed essentially no correlation with attitudes toward criminalization (r = 0.046) and a negative correlation with avoidance of personal contact (r = −0.378). This suggests that knowledge-based interventions alone will be insufficient to address all forms of HIV stigma. As with general vaccination programs, multiple approaches are needed: education about transmission and treatment, direct contact with people living with HIV when possible and appropriate, training for healthcare providers to address their own stigma, and policy-level interventions to remove punitive laws and promote human rights approaches to HIV. Interestingly, healthcare field was not a significant predictor of stigma in our multivariate model, though healthcare professionals and students showed marginally less stigmatizing attitudes in bivariate comparisons. This contrasts with findings from some other studies where healthcare training was associated with lower stigma [17] but aligns with research showing that healthcare workers in the MENA region often harbor significant HIV-related stigma despite their medical knowledge [18,19]. This suggests that medical education in the region may not adequately address HIV stigma, providing valuable insights for medical schools and continuing education programs.

Beaulieu et al. (2014) [9] similarly showed that stigmatizing attitudes toward people living with HIV are multidimensional and require comprehensive interventions that address cognitive, affective, and behavioral components. Multiple studies have shown that interventions combining education, contact, and skills-building are most effective at reducing HIV stigma [13,20]. Our findings highlight that different stigma dimensions may require different intervention strategies: fear-based stigma related to transmission may respond well to education, while moralistic stigma may require more complex interventions that engage with cultural and religious beliefs.

### Limitations

A number of limitations are important to highlight. The use of convenience sampling through social media introduces significant selection bias and limits generalizability, potentially overrepresenting younger, more educated individuals with internet access while underrepresenting other demographic groups. The cross-sectional design limits our ability to establish causal relationships, and our findings reflect associations rather than causation. As with any cross-sectional study, several biases such as social desirability, recall, and response bias can affect results. However, by distributing the survey across all the Emirates as well as ensuring participants’ anonymity helped ensure authentic and genuine responses. We did not collect information about participants’ sexual orientation, HIV testing history, or detailed knowledge about antiretroviral treatment. Because this was a general-population survey, we cannot draw conclusions about stigma experienced by key populations. The regression analysis was also exploratory, and we did not formally assess multicollinearity, residual normality, or homoscedasticity, which may affect the reliability of the estimates.

## Conclusion

This study, the first comprehensive assessment of HIV knowledge and stigma in the UAE, highlights several gaps in knowledge about HIV transmission and widespread stigmatizing attitudes, despite a moderately high overall knowledge score (75.5%). Stigma was particularly pronounced regarding criminalization of transmission and moralistic views linking HIV to moral decline. While knowledge was associated with less stigma, the moderate correlation suggests education alone is insufficient to address deeply rooted attitudes. Overall, the results highlight several actionable recommendations. First, there is a clear need to implement targeted educational campaigns addressing specific misconceptions about HIV transmission, particularly regarding condom effectiveness and casual contact risks. Second, efforts should move away from punitive approaches toward public health and human rights frameworks. Third, healthcare providers need training in providing stigma-free care. Fourth, creating opportunities for positive contact with people living with HIV, where culturally appropriate, may effectively reduce stigma. Finally, the UAE’s high internet penetration and social media engagement present unique opportunities to innovate HIV education through influencer partnerships, mobile applications, and telemedicine platforms. Addressing HIV stigma is critical for achieving UNAIDS 95-95-95 targets and improving HIV prevention and treatment outcomes in the region.

## Supporting information

S1 File Questionnaires. (PDF)

S2 File Graphical abstract. (JPG)

## References

[1] Aldhaleei WA, Bhagavathula AS. HIV/AIDS-knowledge and attitudes in the Arabian Peninsula: A systematic review and meta-analysis. J Infect Public Health. 2020;13(7):939–48. doi: 10.1016/j.jiph.2020.04.002 32359925

[2] Karbasi A, Fordjuoh J, Abbas M, Iloegbu C, Patena J, Adenikinju D, et al. An evolving HIV epidemic in the Middle East and North Africa (MENA) region: A scoping review. Int J Environ Res Public Health. 2023;20(5):3844. doi: 10.3390/ijerph20053844 36900856 PMC10001308

[3] Sharhani A, Jorjoran Shushtari Z, Rahmani A, Armoon B, Noroozi M, Ahounbar E, et al. Incidence of HIV and HCV in people who inject drugs: A systematic and meta-analysis review protocol. BMJ Open. 2021;11(1):e041482. doi: 10.1136/bmjopen-2020-041482 33452193 PMC7813328

[4] Joint United Nations Programme on HIV/AIDS (UNAIDS). Global report: UNAIDS report on the global AIDS epidemic 2013. Geneva: Joint United Nations Programme on HIV/AIDS (UNAIDS); 2013. https://www.unaids.org/en/resources/documents/2013/20130923_UNAIDS_Global_Report_2013

[5] Taha H, Alhawamdeh T, Alkhaldi SM, Ali Albsoul R, Al-Ani A, Awamleh S, et al. Knowledge and attitudes of medical students regarding human papilloma virus infection and vaccine: Cross-sectional study from Jordan. Front Cell Infect Microbiol. 2025;15:1657090. doi: 10.3389/fcimb.2025.1657090 41050749 PMC12488729

[6] Mumtaz GR, Hilmi N, Majed EZ, Abu-Raddad LJ. Characterising HIV/AIDS knowledge and attitudes in the Middle East and North Africa: Systematic review and data synthesis. Glob Public Health. 2020;15(2):275–98. doi: 10.1080/17441692.2019.1668452 31558094

[7] Haghdoost AA, Pourkhandani A, Motaghipisheh SH, Farhoudi B, Fahimifar N, Sadeghirad B. Knowledge and attitude concerning HIV/AIDS among Iranian population: A systematic review and meta-analysis. Iranian J Epidemiol. 2011;6:8–20.

[8] Ramalingam K, Peeran S, Naveen Kumar P, Peeran S, Elhammali NAJ, Elhassan A, et al. Knowledge and attitudes of libyan dental students about HIV/AIDS infection and HIV-positive patients. Dent Med Res. 2015;3(1):8. doi: 10.4103/2348-1471.149566

[9] Beaulieu M, Adrien A, Potvin L, Dassa C, Comité consultatif sur les attitudes envers les PVVIH. Stigmatizing attitudes towards people living with HIV/AIDS: Validation of a measurement scale. BMC Public Health. 2014;14:1246. doi: 10.1186/1471-2458-14-1246 25476441 PMC4289343

[10] Chesney MA, Smith AW. Critical delays in HIV testing and care. Am Behav Scientist. 1999;42(7):1162–74. doi: 10.1177/00027649921954822

[11] Herek GM, Capitanio JP, Widaman KF. HIV-related stigma and knowledge in the United States: Prevalence and trends, 1991–1999. Am J Public Health. 2002;92:371–7. doi: 10.2105/ajph.92.3.371 PMC1447082 11867313

[12] UNAIDS. UNAIDS Fact Sheet 2026: Middle East and North Africa. Joint United Nations Programme on HIV/AIDS; 2026. https://www.unaids.org/en/regionscountries/middleeastandnorthafrica

[13] Stangl AL, Lloyd JK, Brady LM, Holland CE, Baral S. A systematic review of interventions to reduce HIV-related stigma and discrimination from 2002 to 2013: How far have we come?. J Int AIDS Soc. 2013;16(3 Suppl 2):18734. doi: 10.7448/IAS.16.3.18734 24242268 PMC3833106

[14] Nyblade LC. Measuring HIV stigma: Existing knowledge and gaps. Psychol Health Med. 2006;11(3):335–45. doi: 10.1080/13548500600595178 17130069

[15] Allport GW. The nature of prejudice. Cambridge, Mass: Addison-Wesley Pub. Co.; 1954.

[16] Galletly CL, Pinkerton SD. Conflicting messages: How criminal HIV disclosure laws undermine public health efforts to control the spread of HIV. AIDS Behav. 2006;10(5):451–61. doi: 10.1007/s10461-006-9117-3 16804750

[17] Adebajo SB, Bamgbala AO, Oyediran MA. Attitudes of health care providers to persons living with HIV/AIDS in Lagos State, Nigeria. Afr J Reprod Health. 2003;7(1):103–12. doi: 10.2307/3583350 12816317

[18] Attal BA, Al-Rowaishan KM, Akeel AA, AlAmmar FK. HIV stigma in the teaching hospitals in Sana’a, Yemen: A conflict and low-resource setting. BMC Public Health. 2021;21(1):1793. doi: 10.1186/s12889-021-11845-y 34610815 PMC8493713

[19] Aghamolaei T, Tavafian S-S, Hasani L, Zare S. Attitudes of healthcare providers towards patients with HIV/AIDS in Bandar Abbas. Arch Iran Med. 2009;12(3):298–301. 19400609

[20] Brown L, Macintyre K, Trujillo L. Interventions to reduce HIV/AIDS stigma: What have we learned?. AIDS Educ Prev. 2003;15(1):49–69. doi: 10.1521/aeap.15.1.49.23844 12627743

