# Supplementary material for: HIV/AIDS knowledge and stigmatizing attitudes among adult residents of the United Arab Emirates: A cross-sectional study
Source: PLoS One. 2026 Nov 6;21(11):e0362376. doi: 10.1371/journal.pone.0362376 (PMC13742881; doi:10.1371/journal.pone.0362376)
Supplement: Supplementary file 1 [file pone.0362376.s001.pdf]

Stigmatizing attitudes towards HIV/AIDS virus in the United Arab Emirates

We are a group of medical students at the University of Sharjah conducting a research project about the "Stigmatizing attitudes towards HIV/AIDS virus in the United Arab Emirates." The purpose of this study is to evaluate the stigma the general population in the United Arab Emirates has towards people living with HIV/AIDS virus.

You have been selected to participate in this study and your participation is strictly voluntary. If you agree to participate, you will be asked to fill out a questionnaire that should take 5 to 7 minutes of your time. There are no risks associated with participation in this study.

Please note that you must be 18 years or older to participate in this study.

We assure you that your responses will be confidential and will be used only for research purposes. Hence, after submitting your response, it cannot be removed from the database as it is no longer identifiable. There will be no benefits gained from participation in this study.

If you have any questions regarding this study, please feel free to contact our Research Supervisors - Prof. Eman Abu-Gharbieh at eabugharbieh@sharjah.ac.ae or 06-5057289 or Dr. Hiba Barqawi at hbarqawi@sharjah.ac.ae or 06-5057268.

In case of concerns or complaints regarding your participation in this research, you can contact the Research Ethics Committee at: 06-5057304 or by Email: REC@sharjah.ac.ae
Thanks in advance for your participation.

لإجابة الإستبيان باللغة العربية اضغط هنا:
https://forms.gle/Z53pKgUxGwdrmk486

* Indicates required question

1. Please check the box below to proceed to the survey: *

Check all that apply.

- [ ] I have read the above information and agree to participate in this research.
- [ ] I am 18 years or older

2. Do you live in the UAE? *

Mark only one oval.

- ( ) Yes
- ( ) No

Demographics

Please choose the option that best describes you.

3. Sex: * Dropdown

Mark only one oval.

- ( ) Male
- ( ) Female

4. Age: *

____

5. Highest Degree Obtained: * Dropdown

Mark only one oval.

- ( ) Middle School or lower
- ( ) High School
- ( ) Diploma/ Bachelor's Degree
- ( ) Postgraduate Degree (MSc, PhD., etc.) or higher

6. Marital Status: *

Dropdown

Mark only one oval.

- ( ) Single
- ( ) Married
- ( ) Divorced
- ( ) Widowed

7. Nationality: *

Dropdown

Mark only one oval.

- ( ) UAE National
- ( ) Other Arab
- ( ) Non-Arab

8. Place of Residence: *

Dropdown

Mark only one oval.

- ( ) Abu Dhabi
- ( ) Dubai
- ( ) Sharjah
- ( ) Ajman
- ( ) Umm Al Quwain
- ( ) Ras Al Khaimah
- ( ) Fujairah

9. Field of work: *

Dropdown

Mark only one oval.

- ( ) Healthcare (Nurses, Doctors, Dentists, Pharmacists, Healthcare Administration, etc.)
- ( ) Non-Healthcare
- ( ) Student (Health Sciences, Medicine, Dentistry, etc.)
- ( ) Student (Other non-health related majors)
- ( ) Housewife
- ( ) Unemployed (healthcare background)
- ( ) Unemployed (non-healthcare background)

10. Do you have health insurance? *

Dropdown

Mark only one oval.

- ( ) Yes
- ( ) No

For all the questions that follow, please note that HIV is the same as AIDS virus (which is also known as the SIDA virus).

11. Have you heard before about the AIDS virus before? *

Dropdown

Mark only one oval.

- ( ) Yes
- ( ) No

12. How knowledgeable are you about the AIDS virus? *

Mark only one oval.

- Not at all *Skip to question 22*
- Slightly
- Moderately
- Very
- Extremely

13. Do you know anyone with the AIDS virus? * Dropdown

Mark only one oval.

- Yes
- No

AIDS virus transmission

The following questions are related to the AIDS virus and the different ways it can be transmitted

14. Can a healthy-looking person have the AIDS virus? *

Mark only one oval.

- Yes
- No
- I don't know

What is the risk of acquiring HIV through:

15. kissing a person infected with the AIDS virus on the cheek? *

Mark only one oval.

- Very low
- Low
- High
- Very high

16. sharing a glass with a person infected with the AIDS virus? *

Mark only one oval.

- Very low
- Low
- High
- Very high

17. being coughed or sneezed on by a person infected with the AIDS virus? *

Mark only one oval.

- Very low
- Low
- High
- Very high

18. shaking hands with a person infected with the AIDS virus? *

Mark only one oval.

- Very low
- Low
- High
- Very high

19. working in the same office as a person infected with the AIDS virus? *

Mark only one oval.

- Very low
- Low
- High
- Very High

20. having sex with an infected person **using** a condom? *

Mark only one oval.

- Very low
- Low
- High
- Very high

21. having sex with an infected person **without using** a condom? *

Mark only one oval.

- Very low
- Low
- High
- Very high

AIDS virus stigmatizing attitudes

The following questions ask about some of your experiences, feelings, and opinions regarding people living with the AIDS virus.

22. Being around someone who has AIDS does NOT bother me. *

Mark only one oval.

- Strongly disagree
- Disagree
- Agree
- Strongly Agree

23. I would NOT be worried for my health if my coworker had AIDS. *

Mark only one oval.

- Strongly disagree
- Disagree
- Agree
- Strongly agree

24. It would NOT bother me if there was a clinic for people with AIDS on my street. *

Mark only one oval.

- Strongly disagree
- Disagree
- Agree
- Strongly agree

25. I could NOT be friends with someone who has AIDS. *

Mark only one oval.

- Strongly disagree
- Disagree
- Agree
- Strongly agree

26. I would limit my contact with a person whom I know is infected with AIDS. *

Mark only one oval.

- Strongly disagree
- Disagree
- Agree
- Strongly agree

27. I would NOT hug someone with AIDS. *

Mark only one oval.

- Strongly disagree
- Disagree
- Agree
- Strongly agree

28. People who use injectable drugs deserve to have AIDS. *

Mark only one oval.

- Strongly disagree
- Disagree
- Agree
- Strongly agree

29. My support for a person living with AIDS depends on how the person was infected. *

Mark only one oval.

- Strongly disagree
- Disagree
- Agree
- Strongly agree

30. I am disgusted by persons who were infected during homosexual relations. *

Mark only one oval.

- Strongly disagree
- Disagree
- Agree
- Strongly agree

31. To fight AIDS, it is necessary that young people do not have sex. *

Mark only one oval.

- Strongly disagree
- Disagree
- Agree
- Strongly agree

32. Reinforcement of traditional sexual values will help to control AIDS. *

Mark only one oval.

- Strongly disagree
- Disagree
- Agree
- Strongly agree

33. The emergence of AIDS is associated with increased sexual freedom. *

Mark only one oval.

- Strongly disagree
- Disagree
- Agree
- Strongly agree

34. The spread of AIDS is linked to the decline of moral values. *

Mark only one oval.

- Strongly disagree
- Disagree
- Agree
- Strongly agree

35. People who have AIDS should have the right to work serving the public, as waiters-waitresses, cooks, hairdressers, etc. *

Mark only one oval.

- Strongly disagree
- Disagree
- Agree
- Strongly agree

HIV Stigmatizing attitudes (Continued)

The following are *more* questions about some of **your** experiences, feelings, and opinions regarding people living with the AIDS virus.

36. Children who are infected with the AIDS virus should be able to go to daycare. *

Mark only one oval.

- Strongly disagree
- Disagree
- Agree
- Strongly agree

37. Doctors with AIDS should be allowed to go on working with their patients. *

Mark only one oval.

- Strongly disagree
- Disagree
- Agree
- Strongly agree

38. People who are infected with the AIDS virus because they have not used a condom deserve what they get. *

Mark only one oval.

- Strongly disagree
- Disagree
- Agree
- Strongly agree

39. People infected with the AIDS virus should NOT be allowed to live and/or work in the United Arab Emirates. *

Mark only one oval.

- Strongly disagree
- Disagree
- Agree
- Strongly agree

40. If I had a roommate and discovered he/she was infected with the AIDS virus, it would NOT bother me. *

Mark only one oval.

- Strongly disagree
- Disagree
- Agree
- Strongly agree

41. Transmitting the AIDS virus should be punishable by law. *

Mark only one oval.

- Strongly disagree
- Disagree
- Agree
- Strongly agree

42. I have the right to know if someone around me is infected with the AIDS virus. *

Mark only one oval.

- Strongly disagree
- Disagree
- Agree
- Strongly agree

43. People with AIDS have only themselves to blame. *

Mark only one oval.

- Strongly disagree
- Disagree
- Agree
- Strongly agree

44. People who know they are infected with the AIDS virus and who transmit the virus are criminals. *

Mark only one oval.

- Strongly disagree
- Disagree
- Agree
- Strongly agree

45. When a screening test indicates that someone is infected with the AIDS virus, the result should remain confidential. *

Mark only one oval.

- Strongly disagree
- Disagree
- Agree
- Strongly agree

46. Transmitting the AIDS virus is a crime. *

Mark only one oval.

- Strongly disagree
- Disagree
- Agree
- Strongly agree

47. Most people with AIDS are responsible for having their illness. *

Mark only one oval.

- Strongly disagree
- Disagree
- Agree
- Strongly agree

48. Doctors should report the names of people with AIDS to the government. *

Mark only one oval.

- Strongly disagree
- Disagree
- Agree
- Strongly agree

Thank you for your response!

This content is neither created nor endorsed by Google.

Google Forms

وصمة المجتمع تجاه فيروس نقص المناعة المكتسبة/الإيدز في الإمارات العربية المتحدة

نحن مجموعة من طلاب الطب في جامعة الشارقة نقوم بتنفيذ مشروع بحثي حول "وصمة المجتمع تجاه فيروس نقص المناعة البشرية/الإيدز في الإمارات العربية المتحدة". غرض هذه الدراسة هو تقييم التمييز الذي يظهره سكان دولة الإمارات العربية المتحدة تجاه الأشخاص الذين يعيشون مع فيروس نقص المناعة البشرية/الإيدز.

تم اختيارك للمشاركة في هذه الدراسة ومشاركتك تتم على أساس طوعي تماً ما. إذا وافقت على المشاركة، سيُطلب منك ملء استبيان يستغرق ما بين 5 إلى 7 دقائق من وقتك. لا توجد مخاطر مرتبطة بالمشاركة في هذه الدراسة.

للمشاركة في هذه الدراسة، يتطلب أن يكون عمر المشارك 18 عاً ما فأكثر.

نؤكد لكم أن جميع الإجابات ستظل سرية وسيتم استخدامها فقط لأغراض البحث. ومع ذلك، بمجرد تقديم الإجابة، لا يمكن إزالتها من قاعدة البيانات بسبب عدم إمكانية التعرف عليها. يجب التنبيه إلى أنه لن يتم تحقيق أي فوائد شخصية من المشاركة في هذه الدراسة.

إذا كان لديك أي أسئلة بخصوص هذه الدراسة، فلا تتردد في الاتصال بمشرفي البحث - البروفيسورة **إيمان أبو غربية** على البريد الإلكتروني eabugharbieh@sharjah.ac.ae أو على الرقم 06-5057289،
أو الدكتورة **هبة برقاوي** على البريد الإلكتروني hbarqawi@sharjah.ac.ae أو على الرقم 06-5057268.
في حال وجود أي مخاوف أو شكاوى بخصوص مشاركتك في هذا البحث، يمكنك التواصل مع لجنة أخلاقيات البحث على الرقم 06-5057304
او عبر البريد الإلكتروني: REC@sharjah.ac.ae

ونشكرك مقدً ما على مشاركتك.

* Indicates required question

1. يرجى تحديد المربع ادناه لمتابعة الاستبيان: *

Check all that apply.

☐ لقد قمت بقراءة المعلومات السابقة و أوافق على المشاركة في هذا البحث.

2. هل تعيش في الامارات؟ *

Mark only one oval.

- نعم
- لا

البيانات الديموغرافية

3. الجنس * Dropdown

Mark only one oval.

- ذكر
- أنثى

4. العمر *

____

5. المستوى التعليمي * Dropdown

Mark only one oval.

- مدرسة إعدادية أو أقل
- مدرسة ثانوية
- دبلوم مهني
- شهادة جامعية
- دراسات عليا

6. الحالة الاجتماعية *

Mark only one oval.

- متزوج/ متزوجة
- أعزب/ عزباء
- مطلق/ مطلقة
- أرمل/ أرملة
- Other: ____________

7. الجنسية * Dropdown

Mark only one oval.

- اماراتي
- عربي (غير اماراتي)
- غير عربي

8. مكان الإقامة * Dropdown

Mark only one oval.

- أبوظبي
- دبي
- الشارقة
- عجمان
- أم القيوين
- رأس الخيمة
- الفجيرة

9. الوظيفة الحالية *

Mark only one oval.

- عامل في القطاع الصحي (طبيب، ممرض، طبيب أسنان، صيدلاني، إدارة الرعاية الصحية)
- عامل في مجال آخر
- طالب (العلوم الصحية، الطب، طب الأسنان..الخ)
- طالب (تخصص غير طبي)
- ربة منزل
- غير موظف (مع خلفية في مجال الرعاية الصحية)
- غير موظف (بدون خلفية في مجال الرعاية الصحية)
- Other: ______________________

10. هل لديك تأمين صحي؟ * Dropdown

Mark only one oval.

- نعم
- لا

في كل الأسئلة التالية، يرجى ملاحظة أن فيروس نقص المناعة البشرية هو نفسه فيروس الإيدز.

11. هل سمعت من قبل عن فيروس الإيدز؟ *

Mark only one oval.

- نعم
- لا

12. كم تعرف عن فيروس الإيدز؟ *

Mark only one oval.

- لا شيء على الإطلاق — *Skip to question 22*
- القليل
- متوسط
- الكثير
- كل شيء

13. هل تعرف أي شخص مصاب بفيروس الإيدز؟ *

Mark only one oval.

- نعم
- لا

نقل فيروس نقص المناعة البشرية

الأسئلة التالية متعلقة بفيروس نقص المناعة البشرية والطرق المختلفة التي يمكن أن يتم نقله من خلالها.

14. هل يمكن لشخص يبدو بصحة جيدة أن يكون مصابًا بفيروس الإيدز؟ *

Mark only one oval.

- نعم
- لا
- لا اعلم

:ما هو خطر اكتساب فيروس نقص المناعة البشرية من خلال

15. تقبيل شخص مصاب بفيروس الإيدز على الخد؟ *

Mark only one oval.

- منخفض جدا
- منخفض
- عالي
- عالي جدا

16. مشاركة كوب مع شخص مصاب بفيروس الإيدز؟ *

Mark only one oval.

- منخفض جدا
- منخفض
- عالي
- عالي جدا

17. أن يسعل أو يعطس عليك شخص مصاب بفيروس الإيدز؟ *

Mark only one oval.

- منخفض جدا
- منخفض
- عالي
- عالي جدا

18. مصافحة يد شخص مصاب بفيروس الإيدز؟ *

Mark only one oval.

- منخفض جدا
- منخفض
- عالي
- عالي جدا

19. العمل في نفس المكتب مع شخص مصاب بفيروس الإيدز؟ *

Mark only one oval.

- منخفض جدا
- منخفض
- عالي
- عالي جدا

20. ممارسة الجنس **باستخدام** الواقي الذكري مع شخص مصاب بفيروس الإيدز؟ *

Mark only one oval.

- منخفض جدا
- منخفض
- عالي
- عالي جدا

21. * ممارسة الجنس **بدون استخدام** الواقي الذكري مع شخص مصاب بفيروس الإيدز؟

Mark only one oval.

- منخفض جدا
- منخفض
- عالي
- عالي جدا

وصمة المجتمع تجاه فيروس نقص المناعة البشرية.

الأسئلة التالية تتعلق ببعض تجاربك ومشاعرك وآرائك فيما يتعلق بالأشخاص المصابين بفيروس الإيدز.

22. * التواجد حول شخص مصاب بفيروس الإيدز لا يزعجني.

Mark only one oval.

- أختلف تماماً
- أختلف
- أوافق
- أوافق تماماً

23. * لن أكون قلقًا بشأن صحتي إذا كان زميل عملي مصاب بفيروس الإيدز.

Mark only one oval.

- أختلف تماماً
- أختلف
- أوافق
- أوافق تماماً

24. لن يزعجني إذا كان هناك عيادة للأشخاص المصابين بفيروس الإيدز في شارع منزلي. *

Mark only one oval.

- أختلف تماماً
- أختلف
- أوافق
- أوافق تماماً

25. لا يمكنني أن أكون صديقًا لشخص مصاب من فيروس الإيدز. *

Mark only one oval.

- أختلف تماماً
- أختلف
- أوافق
- أوافق تماماً

26. سأقلل من التواصل مع الشخص المصاب بفيروس الإيدز. *

Mark only one oval.

- أختلف تماماً
- أختلف
- أوافق
- أوافق تماماً

27. لن أعانق شخصًا مصابًا بفيروس الإيدز. *

Mark only one oval.

- أختلف تماماً
- أختلف
- أوافق
- أوافق تماماً

28. الأشخاص الذين يحقنون المخدرات يستحقون الإصابة بفيروس الإيدز. *

Mark only one oval.

- أختلف تماماً
- أختلف
- أوافق
- أوافق تماماً

29. دعمي لشخص مصاب بفيروس الإيدز يعتمد على طريقة إصابته بالفيروس. *

Mark only one oval.

- أختلف تماماً
- أختلف
- أوافق
- أوافق تماماً

30. أشعر بالاشمئزاز من الأشخاص الذين أُصيبوا عن طريق العلاقات الجنسية المثلية. *

Mark only one oval.

- أختلف تماماً
- أختلف
- أوافق
- أوافق تماماً

31. لمكافحة فيروس الايدز، من الضروري أن لا يمارس الشباب الجنس. *

Mark only one oval.

- أختلف تماماً
- أختلف
- أوافق
- أوافق تماماً

32. تعزيز القيم الجنسية التقليدية سيساعد في السيطرة على فيروس الإيدز. *

Mark only one oval.

- أختلف تماماً
- أختلف
- أوافق
- أوافق تماماً

33. ظهور فيروس الإيدز مرتبط بزيادة حرية الممارسة الجنسية. *

Mark only one oval.

- أختلف تماماً
- أختلف
- أوافق
- أوافق تماماً

34. انتشار فيروس الإيدز مرتبط بانخفاض القيم الأخلاقية. *

Mark only one oval.

- أختلف تماماً
- أختلف
- أوافق
- أوافق تماماً

35. يجب أن يحق للأشخاص المصابون بفيروس الإيدز أن يعملوا في وظائف ذات صلة بالمجتمع مثل النوادل و الطهاة و الحلاقين و غيرها. *

Mark only one oval.

- أختلف تماماً
- أختلف
- أوافق
- أوافق تماماً

"مواقف المجتمع المعيبة تجاه فيروس نقص المناعة البشرية "متابعة

التالي: المزيد من الأسئلة حول بعض تجاربك ومشاعرك وآرائك فيما يتعلق بالأشخاص الذين يعيشون مع فيروس الإيدز.

36. ينبغي أن يُسمح للأطفال المصابين بفيروس الإيدز بالحضور إلى دور الحضانة. *

Mark only one oval.

- أختلف تماماً
- أختلف
- أوافق
- أوافق تماماً

37. يجب السماح للأطباء المصابين بفيروس الإيدز بمواصلة العمل مع مرضاهم. *

Mark only one oval.

- أختلف تماماً
- أختلف
- أوافق
- أوافق تماماً

38. الأشخاص الذين اصيبوا بفيروس الإيدز الذين لم يستخدموا الواقي الذكري ما يستحقون ما اصابهم . *

Mark only one oval.

- أختلف تماماً
- أختلف
- أوافق
- أوافق تماماً

39. لا يجب السماح للأشخاص المصابين بفيروس الإيدز بالاقامة و/أو العمل في دولة الإمارات العربية المتحدة. *

Mark only one oval.

- أختلف تماماً
- أختلف
- أوافق
- أوافق تماماً

40. إذا اكتشفت أن زميل السكن لدي مصاب بفيروس الإيدز فلن يزعجني ذلك. *

Mark only one oval.

- أختلف تماماً
- أختلف
- أوافق
- أوافق تماماً

41. يجب أن يكون لنقل فيروس الإيدز عقابًا قانونيًا. *

Mark only one oval.

- أختلف تماماً
- أختلف
- أوافق
- أوافق تماماً

42. .لدي الحق في معرفة ما إذا كان الشخص حولي مصابًا بفيروس الإيدز *

Mark only one oval.

- أختلف تماماً
- أختلف
- أوافق
- أوافق تماماً

43. .الأشخاص المصابون بفيروس الإيدز لا يمكنهم لوم غير أنفسهم *

Mark only one oval.

- أختلف تماماً
- أختلف
- أوافق
- أوافق تماماً

44. .تعتبر جريمة من الأشخاص الذين يعلمون أنهم مصابون بفيروس الإيدز وينقلون الفيروس للاخرين *

Mark only one oval.

- أختلف تماماً
- أختلف
- أوافق
- أوافق تماماً

45. عند اكتشاف أن الشخص مصاب بفيروس الإيدز، ينبغي أن تبقى نتيجة المسح الطبي سرية. *

Mark only one oval.

- أختلف تماماً
- أختلف
- أوافق
- أوافق تماماً

46. يعتبر نقل فيروس الإيدز جريمة. *

Mark only one oval.

- أختلف تماماً
- أختلف
- أوافق
- أوافق تماماً

47. معظم الأشخاص المصابون بفيروس الإيدز مسؤولون عن إصابتهم بالمرض. *

Mark only one oval.

- أختلف تماماً
- أختلف
- أوافق
- أوافق تماماً

48. ينبغي على الأطباء الإبلاغ عن حالات الأشخاص المصابين بفيروس الإيدز إلى الجهات المختصة *

Mark only one oval.

- أختلف تماماً
- أختلف
- أوافق
- أوافق تماماً

شكرا لمشاركتك بالاستبيان
