# Supplementary material for: HIV/AIDS knowledge and stigmatizing attitudes among adult residents of the United Arab Emirates: A cross-sectional study
Source: PLoS One. 2026 Nov 6;21(11):e0362376. doi: 10.1371/journal.pone.0362376 (PMC13742881; doi:10.1371/journal.pone.0362376)
Supplement: Supplementary file 2 [file pone.0362376.s003.docx]

**Stigmatizing attitudes and knowledge of HIV/AIDS in the United Arab Emirates**

Title-Incomplete, as the study population is not provided

Abstract-Well written

Methodology – remove the full form of UAE from the study design, as it is already stated, and use only abbreviations

The social media platform was utilised as a channel for recruiting participants. How did the authors ensure that the participants were UAE residents? State it in the methodology

Clearly specify the inclusion and exclusion criteria

The terms Awareness and knowledge were used interchangeably. Please use the term knowledge throughout the manuscript so that it reflects the title.

Rationalise the reason for piloting on 20 people

Specify how the level of knowledge was scored into good or poor, adequate or inadequate level of knowledge

Same with the stigmatization attitude, how the scoring was done, and how positive and negative attitudes were ascertained.

Remarks: Minor corrections
