# Supplementary material for: HIV/AIDS knowledge and stigmatizing attitudes among adult residents of the United Arab Emirates: A cross-sectional study
Source: PLoS One. 2026 Nov 6;21(11):e0362376. doi: 10.1371/journal.pone.0362376 (PMC13742881; doi:10.1371/journal.pone.0362376)
Supplement: Supplementary file 3 [file pone.0362376.s004.docx]

**REVIEWER REPORT**

**Critical Appraisal** of the manuscript **PONE-D-26-06560**, entitled "Stigmatizing attitudes and knowledge of HIV/AIDS in the United Arab Emirates"

**Strengths**

The manuscript addresses an important and relevant public health issue and contributes evidence from a setting where data remain relatively limited. It is well contextualized within the existing literature, with a clear rationale and objectives. Also, the use of a large sample size and a validated HIV stigma assessment tool strengthens the study. The analytical approach is generally appropriate, clearly described, and sufficiently detailed to support reproducibility. The manuscript is well organized, transparently reports its findings, and presents a thoughtful limitations section that acknowledges key study constraints, including those related to convenience sampling and potential selection bias.

**Issues to Address**

While the study is generally well conducted, several aspects of the manuscript would require some revision to enhance methodological transparency, reporting consistency, and overall clarity. Particularly, attention is needed regarding the reporting of study procedures, consistency across manuscript sections, description of analytical decisions, and a few formatting issues. Addressing the comments below would strengthen the rigor and interpretability of the manuscript.

**Table: Summary of Reviewer Comments**

| **Section** | **Verdict** | **Comment/Recommendation** |
| --- | --- | --- |
| 1. Title | Adequate | -The title is clear and reflects the study objectives. No major concerns. |
| 2. Abstract | Clarification required | -Clarify the final analytical sample size if different # of participants were included in different analyses. |
| 3. Introduction | Minor revision required | -Cite the source of the estimate of 38.4 million people living with HIV worldwide.  -Line 4/5: Report the prevalence and cite the source.  -Update UNAIDS statistics using the most recent available data. |
| 4. Methods | Major revision required | -Clarify the consent procedure: The methods section indicates “implied consent”, whereas the Ethics Declaration states that “written informed consent” was obtained.  -The final sample size used in the analysis should be mentioned/introduced in the methods section after the originally calculated minimum sample size.  -Complete ethics statement. It appears incomplete |
| 5. Ethics Declaration | Clarification required | -Inconsistency on “consent” type obtained: Double-check with the methods section |
| 6. Statistical Analysis | Minor revision required  (Optional) | -Justify model selection and report assessment of regression assumptions. |
| 7. Results | Clarification required | -Explain the reduction from N=1,211 to 1171 participants in HIV knowledge and stigma analyses |
| 8. Discussion | Minor revision required | -Look out for wording that suggests representativeness of the UAE population, given the convenience sampling technique used.  -Clarify or remove statements on KMO and Bartlett’s test. These were not described or mentioned in the methods/results sections. |
| 9. Conclusion | Adequate | -Supported by data. No major concerns. |
| 10. Tables | Minor revision required | -For all 4 tables, put the title above the table, not below  -Remove repeated denominators and report N as a footnote below each table (1 & 3) |
| 11. Figures | Minor revision required | -Figures 1 and 2 are duplicated in the submission files. Delete duplicates. |

**In-depth Explanations of Selected Comments**

**Introduction**

- Line 4/5: “The MENA region has one of the lowest reported HIV prevalence…” 🡪 Put the prevalence in brackets and cite the source at the end of the sentence.
- Paragraph 3, Line 3/4/5: Consider updating it to the latest UNAIDS data. E.g., “Access to… remain below the global targets, with **x%** of PLHIV knowing their status and **y%** having access to ART”. Given that the current statistics fall below the 95-95-95 targets, your argument is still maintained

**Methods**

- Study design sub-section: After reporting the minimum sample size (N=385), clearly state how many participants were finally included in the study/analyses.
- Ethics statement language fix: From “University of Sharjah Research Ethics Committee (Reference number: REC-24-02-14-01-F)” to “Ethical approval was obtained from the….”

**Results**

- There’s a sudden denominator change from 1,211 participants in the demographic analyses to 1,171 participants in the HIV knowledge and stigma analyses. That brings N to less than 40. Please clarify the reason for the exclusion of these 40 participants.

**Tables 1 and 3**

- Given that the sample size is consistent across the table (N=1211, for Table 1 and N= 1171 for Table 3), remove /1211 or /1171 from the corresponding table. Rather keep it as a footnote, e.g., Sample Size, N = 1211 or 1171.
- Given the labeled categories, e.g., Disagree % (n), the column content should be **x%(y),** no need to write x% (**n**=y/**N**). E.g. In Table 1, instead of Female, 60.2% (n = 729/1,211), write **60.2% (729):** do the same for the whole table and same for Table 3, after ensuring **N is reported below the table as a footnote.**

**Reporting Issues**

- OLS Regression was performed; however, there is no discussion of the regression assumptions (e.g., multicollinearity, normality of residuals, and homoscedasticity). Consider including a justification statement about why the model was appropriate.
- The discussion mentions factor analysis findings (including KMO and Bartlett's test statistics), yet these analyses are not described in the Methods section nor reported in the Results. Consider reporting the factor analysis methodology and findings appropriately or remove these statements.
- The study reports 1,211 participants, but most of the analyses use N=1171. A simple participant flow/explanation is needed (Questions that could guide this: how many started? How many completed? How many were excluded/why?)
