# Supplementary material for: HIV/AIDS knowledge and stigmatizing attitudes among adult residents of the United Arab Emirates: A cross-sectional study
Source: PLoS One. 2026 Nov 6;21(11):e0362376. doi: 10.1371/journal.pone.0362376 (PMC13742881; doi:10.1371/journal.pone.0362376)
Supplement: Supplementary file 4 [file pone.0362376.s006.docx]

Reviewer 1

Stigmatizing attitudes and knowledge of HIV/AIDS in the United Arab Emirates

Title-Incomplete, as the study population is not provided

Thank you for this comment. We revised the title to identify the study population and study design more clearly. The revised title is: “HIV/AIDS knowledge and stigmatizing attitudes among adult residents of the United Arab Emirates: A cross-sectional study.

Abstract-Well written

Methodology – remove the full form of UAE from the study design, as it is already stated, and use only abbreviations

Thank you. We removed the repeated expansion of the abbreviation and now use “UAE” in the Study Design subsection.

The social media platform was utilised as a channel for recruiting participants. How did the authors ensure that the participants were UAE residents? State it in the methodology

Thank you for raising this point. We clarified that UAE residency was assessed through a mandatory screening question at the beginning of the questionnaire. Participants were required to confirm that they currently lived in the UAE before proceeding with the survey. Only those who answered “Yes” were eligible for inclusion.

Clearly specify the inclusion and exclusion criteria

Thank you. We added explicit inclusion and exclusion criteria to the Methods section. Eligible participants were adults aged 18 years or older who currently resided in the UAE and provided electronic informed consent. Respondents who did not meet these requirements were ineligible. We also clarified that participants without analyzable data for a specific outcome were excluded only from the corresponding outcome analysis.

The terms Awareness and knowledge were used interchangeably. Please use the term knowledge throughout the manuscript so that it reflects the title.

Thank you. We standardized the terminology throughout the manuscript. The term “knowledge” is now used when referring to participants’ understanding of HIV transmission and prevention. The term “previous familiarity” is used only when reporting whether participants had previously heard of HIV/AIDS.

Rationalise the reason for piloting on 20 people

Thank you. We clarified that the pilot involving 20 participants was conducted as a pragmatic pretest to evaluate item clarity, cultural appropriateness, bilingual conceptual equivalence, questionnaire flow, and completion time. It was not intended as a formal psychometric validation study. Minor revisions were made based on the pilot feedback.

Specify how the level of knowledge was scored into good or poor, adequate or inadequate level of knowledge

Thank you. We expanded the description of the HIV knowledge scoring procedure. The seven transmission-risk items were scored so that higher values represented responses closer to the prespecified correct risk level. The item scores were averaged and converted to a 0–100 composite score. Knowledge was analyzed as a continuous variable; therefore, participants were not classified into good, poor, adequate, or inadequate knowledge categories. This has now been stated explicitly in the Methods and Results.

Same with the stigmatization attitude, how the scoring was done, and how positive and negative attitudes were ascertained

Thank you. We clarified the scoring and interpretation of the stigma scale. Positively worded items were coded from 1 for “strongly disagree” to 4 for “strongly agree,” while stigmatizing items were reverse-coded so that higher values consistently represented less stigma. Mean scores were calculated for the theoretical dimensions and the overall scale. Stigma was analyzed as a continuous variable, and no cutoff was used to classify attitudes as positive or negative.

Reviewer 2

**Critical Appraisal** of the manuscript **PONE-D-26-06560**, entitled "Stigmatizing attitudes and knowledge of HIV/AIDS in the United Arab Emirates"

**Strengths**

The manuscript addresses an important and relevant public health issue and contributes evidence from a setting where data remain relatively limited. It is well contextualized within the existing literature, with a clear rationale and objectives. Also, the use of a large sample size and a validated HIV stigma assessment tool strengthens the study. The analytical approach is generally appropriate, clearly described, and sufficiently detailed to support reproducibility. The manuscript is well organized, transparently reports its findings, and presents a thoughtful limitations section that acknowledges key study constraints, including those related to convenience sampling and potential selection bias.

**Issues to Address**

While the study is generally well conducted, several aspects of the manuscript would require some revision to enhance methodological transparency, reporting consistency, and overall clarity. Particularly, attention is needed regarding the reporting of study procedures, consistency across manuscript sections, description of analytical decisions, and a few formatting issues. Addressing the comments below would strengthen the rigor and interpretability of the manuscript.

**Table: Summary of Reviewer Comments**

| **Section** | **Verdict** | **Comment/Recommendation** |
| --- | --- | --- |
| 1. Title | Adequate | -The title is clear and reflects the study objectives. No major concerns. |
| 2. Abstract | Clarification required | -Clarify the final analytical sample size if different # of participants were included in different analyses. |
| 3. Introduction | Minor revision required | -Cite the source of the estimate of 38.4 million people living with HIV worldwide.  -Line 4/5: Report the prevalence and cite the source.  -Update UNAIDS statistics using the most recent available data. |
| 4. Methods | Major revision required | -Clarify the consent procedure: The methods section indicates “implied consent”, whereas the Ethics Declaration states that “written informed consent” was obtained.  -The final sample size used in the analysis should be mentioned/introduced in the methods section after the originally calculated minimum sample size.  -Complete ethics statement. It appears incomplete |
| 5. Ethics Declaration | Clarification required | -Inconsistency on “consent” type obtained: Double-check with the methods section |
| 6. Statistical Analysis | Minor revision required  (Optional) | -Justify model selection and report assessment of regression assumptions. |
| 7. Results | Clarification required | -Explain the reduction from N=1,211 to 1171 participants in HIV knowledge and stigma analyses |
| 8. Discussion | Minor revision required | -Look out for wording that suggests representativeness of the UAE population, given the convenience sampling technique used.  -Clarify or remove statements on KMO and Bartlett’s test. These were not described or mentioned in the methods/results sections. |
| 9. Conclusion | Adequate | -Supported by data. No major concerns. |
| 10. Tables | Minor revision required | -For all 4 tables, put the title above the table, not below  -Remove repeated denominators and report N as a footnote below each table (1 & 3) |
| 11. Figures | Minor revision required | -Figures 1 and 2 are duplicated in the submission files. Delete duplicates. |

**In-depth Explanations of Selected Comments**

**Introduction**

- Line 4/5: “The MENA region has one of the lowest reported HIV prevalence…” 🡪 Put the prevalence in brackets and cite the source at the end of the sentence.
- Paragraph 3, Line 3/4/5: Consider updating it to the latest UNAIDS data. E.g., “Access to… remain below the global targets, with **x%** of PLHIV knowing their status and **y%** having access to ART”. Given that the current statistics fall below the 95-95-95 targets, your argument is still maintained

**Methods**

- Study design sub-section: After reporting the minimum sample size (N=385), clearly state how many participants were finally included in the study/analyses.
- Ethics statement language fix: From “University of Sharjah Research Ethics Committee (Reference number: REC-24-02-14-01-F)” to “Ethical approval was obtained from the….”

**Results**

- There’s a sudden denominator change from 1,211 participants in the demographic analyses to 1,171 participants in the HIV knowledge and stigma analyses. That brings N to less than 40. Please clarify the reason for the exclusion of these 40 participants.

**Tables 1 and 3**

- Given that the sample size is consistent across the table (N=1211, for Table 1 and N= 1171 for Table 3), remove /1211 or /1171 from the corresponding table. Rather keep it as a footnote, e.g., Sample Size, N = 1211 or 1171.
- Given the labeled categories, e.g., Disagree % (n), the column content should be **x%(y),** no need to write x% (**n**=y/**N**). E.g. In Table 1, instead of Female, 60.2% (n = 729/1,211), write **60.2% (729):** do the same for the whole table and same for Table 3, after ensuring **N is reported below the table as a footnote.**

**Reporting Issues**

- OLS Regression was performed; however, there is no discussion of the regression assumptions (e.g., multicollinearity, normality of residuals, and homoscedasticity). Consider including a justification statement about why the model was appropriate.
- The discussion mentions factor analysis findings (including KMO and Bartlett's test statistics), yet these analyses are not described in the Methods section nor reported in the Results. Consider reporting the factor analysis methodology and findings appropriately or remove these statements.
- The study reports 1,211 participants, but most of the analyses use N=1171. A simple participant flow/explanation is needed (Questions that could guide this: how many started? How many completed? How many were excluded/why?)

1- Abstract

Thank you for highlighting this reporting inconsistency. We revised the Abstract to distinguish between the total sample and the analytical sample used for the HIV knowledge and stigma outcomes. The demographic analyses included 1,211 participants, while the HIV knowledge and stigma analyses included 1,171 participants with analyzable outcome data.

2- Introduction
A- Thank you. We updated the global estimate using the latest available UNAIDS data and added the corresponding reference. The revised Introduction now reports that approximately 40.9 million people worldwide were living with HIV in 2025.
B- Thank you. We added the estimated HIV prevalence for the MENA region and cited the latest UNAIDS regional profile. The revised text reports an estimated prevalence of 0.07% among adults aged 15–49 years in 2024.
C- Thank you. We updated the regional epidemiological and HIV treatment-cascade statistics using the latest available UNAIDS data. The manuscript now reports that, in 2024, approximately 63% of people living with HIV in the MENA region knew their status, 48% were receiving antiretroviral therapy, and 44% had achieved viral suppression.
3- Methods
A- Thank you for identifying this inconsistency. Participants did not provide merely implied consent or a physically signed written consent form. They were required to read the electronic participant information sheet and select a mandatory checkbox confirming their agreement to participate before accessing the questionnaire. We have therefore revised both the Methods and Ethics Declaration to use the accurate term “electronic informed consent.”
B- Thank you. We revised the sample-size paragraph and now report both the calculated minimum sample size and the final analytical samples. The minimum required sample was 385 participants. The demographic analyses included 1,211 participants, while the HIV knowledge and stigma analyses included 1,171 participants.
C- Thank you. We completed the ethics statement by identifying the approving committee, approval date, reference number, consent procedure, confidentiality protections, and compliance with the approved study protocol
4- Ethics Declaration

Thank you. We corrected the Ethics Declaration so that it is consistent with the actual consent procedure and the revised Methods section. The manuscript now consistently states that electronic informed consent was obtained from all participants. Thank you for identifying the unexplained change in denominator.

5- Statistical Analysis

Thank you for this comment. We have clarified in the Statistical Analysis section that the regression model was exploratory, intended to identify candidate predictors rather than test a confirmatory model, and we now explicitly state that formal assessment of regression assumptions (multicollinearity, normality of residuals, homoscedasticity) was not performed. A corresponding note has been added to the Limitations paragraph of the Discussion

6- Results

We added a participant-flow explanation in both the Methods and Results. Of the 1,211 participants included in the demographic analyses, 40 did not have complete analyzable HIV knowledge and stigma outcome data, leaving 1,171 participants in the corresponding analyses

7- Discussion
A- Thank you. We revised wording throughout the Discussion to avoid implying national representativeness. The manuscript now consistently refers to the study participants or surveyed sample and explicitly notes that the convenience-sampling method and demographic composition limit generalizability to the wider UAE population.
B- Thank you for identifying this inconsistency. No factor analysis was conducted in this study, and the statements referencing KMO and Bartlett's test did not correspond to any analysis described in the Methods or reported in the Results. These statements have been removed from the Discussion.
8- Conclusion
9- Tables
A- Thank you. The titles of all four tables have been moved above their respective tables.
B- Thank you. We removed repeated denominators from Tables 1 and 3 and now report values in the format “percentage (count).” The applicable sample size is reported in a note below each table.
