# Supplementary material for: HIV/AIDS knowledge and stigmatizing attitudes among adult residents of the United Arab Emirates: A cross-sectional study
Source: PLoS One. 2026 Nov 6;21(11):e0362376. doi: 10.1371/journal.pone.0362376 (PMC13742881; doi:10.1371/journal.pone.0362376)
Supplement: Supplementary file 5 [file pone.0362376.s007.docx]

Dear Editorial Board,

We would like to thank you and the reviewers for taking the time to review our paper (Manuscript ID PONE-D-26-06560R1) titled “HIV/AIDS knowledge and stigmatizing attitudes among adult residents of the United Arab Emirates: A cross-sectional study”, which was submitted to PLOS One. We appreciate your expertise and constructive feedback and hope to address it in an effort to make the paper more comprehensive. Revisions to the manuscript and replies to reviewers’ comments are outlined below.

Reviewer #3: The manuscript has improved substantially, and most of my previous concerns regarding sample size reporting, denominator discrepancies, updated epidemiological information, discussion framing, and table formatting have been satisfactorily addressed. The clarification regarding regression assumptions and the explanation for the reduction from N=1211 to N=1171 have also been adequately addressed.

We thank the reviewer for their careful reassessment and positive evaluation of our revisions. We appreciate the acknowledgment that the major methodological and reporting concerns have been satisfactorily addressed.

A minor inconsistency remains in the description of the consent process. The Methods section refers to electronic informed consent, whereas the Declarations section refers to written informed consent. While the authors may wish to harmonize this wording for clarity and consistency, both sections clearly indicate that informed consent was obtained from participants. In my view, this discrepancy does not raise concerns regarding the ethical conduct of the study and does not affect the scientific rigor, validity, or interpretation of the findings.

We agree and have standardized the description as “electronic informed consent.” We added a corresponding ethics-approval and informed-consent statement under Declarations, consistent with the online consent procedure described in Methods.

Overall, I am satisfied that the authors have adequately addressed the substantive issues raised in the previous review, and I believe the manuscript is suitable for publication.

Reviewer #4: Overall comment

The authors have addressed most of the previous reviewer comments and have substantially improved the clarity and reporting of the manuscript. Two points remain to be addressed (the statistical results in Table 4 and the description of informed consent), and I would encourage the authors to resolve these before the manuscript is considered ready for publication.

Major comment

1. Statistical reporting in Table 4:

I noticed a potential discrepancy in the degrees of freedom reported for the regression model. The analysis includes 1,171 participants and 10 predictors, yet Table 4 reports F(10, 1,171) = 39.97. Given n = 1,171 and k = 10, the residual degrees of freedom would be expected to be 1,160 rather than 1,171. I would ask the authors to verify the degrees of freedom, the F-statistic, and the corresponding R² against the original regression output and correct them if necessary.

Thank you for your comment. We have fixed the degrees of freedom in the table. The python package used does not calculate the degrees of freedom and it has to be calculated manually. The remainder of results remain as is

Minor comment

1. Consent statement:

The description of informed consent appears inconsistent across the manuscript. The Methods section states that electronic informed consent was obtained, whereas the Ethics Statement refers to “informed written consent.” I would ask the authors to clarify which term accurately reflects the procedure and to use consistent terminology throughout the manuscript.

Thank you for highlighting this inconsistency. The questionnaire required participants to affirm agreement electronically before entering the survey. We have used “electronic informed consent” consistently in the revision and added that terminology to the Declarations section.

Reviewer #5: This is a well-written study that provides relevant insights into the region, particularly given the increase in the number of cases reported. The findings are valuable in helping to better understand the regional context, especially in light of the limited data available for this particular region. The added value of the study is clear, and it provides concrete recommendations that are grounded in the findings.

Abstract – well written and clear

Introduction – well written and comprehensive covering importance context and clarifying the value add of this study

- “misconceptions are prevalent (Mumtaz et al., 2020)” – insight on the types of misconceptions captured?

We agree that the description was overly general. We have clarified the types of gaps identified in the regional synthesis, specifically knowledge of transmission modes and perceived risk. The Results and Discussion also give study-specific examples involving casual contact and condom-protected sex

- “despite some declining trends in recent years (Mumtaz et al., 2020)” – any reasons available to explain this decline?

Thank you. The referenced synthesis establishes persistent negative attitudes but does not provide sufficient evidence here to attribute changes over time to a specific cause. To avoid an unsupported temporal claim or speculative explanation, we removed the ambiguous “declining trends” statement.

- “Stigma has profound implications for HIV prevention and treatment, acting as a barrier to testing, disclosure, and care-seeking behaviors.” – the mental health aspect at an individual level may also important to acknowledge, I think that’s missing in the introduction and the discussion

We thank the reviewer for this valuable suggestion. We agree that HIV-related stigma can have important psychological consequences for people living with HIV. However, the primary aim of our study was to assess HIV-related knowledge and stigmatizing attitudes among adult residents of the UAE, rather than the psychological experiences of people living with HIV. As mental health outcomes were not assessed in our study, we believe that a detailed discussion of these effects would extend beyond the scope of our research. The Discussion already acknowledges broader consequences of stigma, including reduced quality of life and social isolation. We therefore prefer to maintain the current focus of the manuscript while recognizing mental health as an important area for future research.

Methods – clear and presented with all necessary information

- “Minor wording and formatting revisions were made based on participant feedbackf.” – just a minor spelling error

Thank you for noting the typo. The sentence has been corrected and clarified.

- During the pilot, did participants have an opportunity to provide feedback on the survey questions, including suggesting additional questions or identifying any questions that could potentially be removed or revised?

We thank the reviewer for this comment. As described in the Methods section, both language versions of the questionnaire were pilot tested with 20 participants to assess clarity, cultural appropriateness, conceptual equivalence, questionnaire flow, and approximate completion time. Minor wording and formatting revisions were made based on participant feedback. We believe the current description adequately reflects the purpose and outcomes of the pilot testing.

- How were the pilot participants selected?

We thank the reviewer for this comment. The 20 pilot participants were randomly selected members of the public who voluntarily participated in the pilot testing. Their feedback was used to assess the clarity, wording, and overall usability of the questionnaire before its distribution.

Results and Discussion

- The results are clearly described and well discussed in the context of the literature in the discussion.

- It’s very useful that the authors identify several actionable recommendations

- One aspect that may be worth considering, depending on its relevance to the discussion, is how the stigma findings relate to the concentration of HIV among key populations, as noted in the introduction. It could be useful to reflect on whether this concentration may also intersect with broader social and cultural perceptions or stigma associated with these populations, and how this might influence the findings on HIV-related stigma.

We agree. We added a cautious interpretation of how HIV-related stigma may overlap with attitudes about sexual behavior, substance use, and key populations. The discussion uses the observed response distribution for one stigma item and clarifies that respondents’ identities, experiences, and intersections of stigma were not measured, so no subgroup-specific inference is made.
