# Supplementary material for: HIV/AIDS knowledge and stigmatizing attitudes among adult residents of the United Arab Emirates: A cross-sectional study
Source: PLoS One. 2026 Nov 6;21(11):e0362376. doi: 10.1371/journal.pone.0362376 (PMC13742881; doi:10.1371/journal.pone.0362376)
Supplement: Peer review history

# Peer review: Decision Letter 0

**DOI:** 10.1371/journal.pone.0362376.r001

**Authors:** Daniel Demant

8 Jul 2026

Dear Dr. Abu Gharbieh,

Thank you for submitting your manuscript to PLOS ONE. After careful consideration, we feel that it has merit but does not fully meet PLOS ONE’s publication criteria as it currently stands. Therefore, we invite you to submit a revised version of the manuscript that addresses the points raised during the review process.

The reviewers recognise that the study addresses an important and underexplored public health issue. However, they identified a number of substantive issues that require attention before the manuscript can be considered further for publication. I agree with the reviewers that revisions are needed to improve methodological transparency, reporting consistency, statistical reporting, interpretation of findings, scoring and validation of the knowledge and stigma measures as well as sampling limitations. Several reviewers also requested clearer descriptions of the questionnaire development, recruitment procedures, ethics and consent processes, and a more careful interpretation of findings consistent with the cross-sectional design. Accordingly, substantial revisions are required before I can consider publication of the manuscript.

Please submit your revised manuscript by Aug 22 2026 11:59PM. If you will need more time than this to complete your revisions, please reply to this message or contact the journal office at plosone@plos.org. When you're ready to submit your revision, log on to https://www.editorialmanager.com/pone/ and select the 'Submissions Needing Revision' folder to locate your manuscript file.

- A letter that responds to each point raised by the academic editor and reviewer(s). You should upload this letter as a separate file labeled 'Response to Reviewers'.
- A marked-up copy of your manuscript that highlights changes made to the original version. You should upload this as a separate file labeled 'Revised Manuscript with Track Changes'.
- An unmarked version of your revised paper without tracked changes. You should upload this as a separate file labeled 'Manuscript'.

If you would like to make changes to your financial disclosure, please include your updated statement in your cover letter. Guidelines for resubmitting your figure files are available below the reviewer comments at the end of this letter.

If applicable, we recommend that you deposit your laboratory protocols in protocols.io to enhance the reproducibility of your results. Protocols.io assigns your protocol its own identifier (DOI) so that it can be cited independently in the future. For instructions see: https://journals.plos.org/plosone/s/submission-guidelines#loc-laboratory-protocols. Additionally, PLOS ONE offers an option for publishing peer-reviewed Lab Protocol articles, which describe protocols hosted on protocols.io. Read more information on sharing protocols at https://plos.org/protocols?utm_medium=editorial-email&utm_source=authorletters&utm_campaign=protocols.

As the corresponding author, your ORCID iD is verified in the submission system and will appear in the published article. PLOS supports the use of ORCID, and we encourage all coauthors to register for an ORCID iD and use it as well. Please encourage your coauthors to verify their ORCID iD within the submission system before final acceptance, as unverified ORCID iDs will not appear in the published article. *Only*  the individual author can complete the verification step; PLOS staff *cannot*  verify ORCID iDs on behalf of authors.

We look forward to receiving your revised manuscript.

Kind regards,

Daniel Demant, PhD

Academic Editor

PLOS One

Journal Requirements:

When submitting your revision, we need you to address these additional requirements.

1. Please ensure that your manuscript meets PLOS ONE’s style requirements, including those for file naming. The PLOS ONE style templates can be found at

https://journals.plos.org/plosone/s/file?id=wjVg/PLOSOne_formatting_sample_main_body.pdf and

https://journals.plos.org/plosone/s/file?id=ba62/PLOSOne_formatting_sample_title_authors_affiliations.pdf

2. We note that your Data Availability Statement is currently as follows: All relevant data are within the manuscript and its Supporting Information files.

Please confirm at this time whether or not your submission contains all raw data required to replicate the results of your study. Authors must share the “minimal data set” for their submission. PLOS defines the minimal data set to consist of the data required to replicate all study findings reported in the article, as well as related metadata and methods (https://journals.plos.org/plosone/s/data-availability#loc-minimal-data-set-definition).

For example, authors should submit the following data:

- The values behind the means, standard deviations and other measures reported;

- The values used to build graphs;

- The points extracted from images for analysis.

Authors do not need to submit their entire data set if only a portion of the data was used in the reported study.

If your submission does not contain these data, please either upload them as Supporting Information files or deposit them to a stable, public repository and provide us with the relevant URLs, DOIs, or accession numbers. For a list of recommended repositories, please see https://journals.plos.org/plosone/s/recommended-repositories.

If there are ethical or legal restrictions on sharing a de-identified data set, please explain them in detail (e.g., data contain potentially sensitive information, data are owned by a third-party organization, etc.) and who has imposed them (e.g., an ethics committee). Please also provide contact information for a data access committee, ethics committee, or other institutional body to which data requests may be sent. If data are owned by a third party, please indicate how others may request data access.

3. Please include captions for your Supporting Information files at the end of your manuscript, and update any in-text citations to match accordingly. Please see our Supporting Information guidelines for more information: http://journals.plos.org/plosone/s/supporting-information.

4. If the reviewer comments include a recommendation to cite specific previously published works, please review and evaluate these publications to determine whether they are relevant and should be cited. There is no requirement to cite these works unless the editor has indicated otherwise.

[Note: HTML markup is below. Please do not edit.]

Reviewers' comments:

Reviewer’s Responses to Questions

**Comments to the Author**

1. Is the manuscript technically sound, and do the data support the conclusions?

Reviewer #1: Yes

Reviewer #2: Partly

Reviewer #3: Yes

Reviewer #4: Partly

Reviewer #5: Yes

**********

2. Has the statistical analysis been performed appropriately and rigorously?

Reviewer #1: Yes

Reviewer #2: Yes

Reviewer #3: Yes

Reviewer #4: No

Reviewer #5: Yes

**********

3. Have the authors made all data underlying the findings in their manuscript fully available?

The PLOS Data policy

Reviewer #1: Yes

Reviewer #2: No

Reviewer #3: Yes

Reviewer #4: No

Reviewer #5: Yes

**********

4. Is the manuscript presented in an intelligible fashion and written in standard English?

Reviewer #1: Yes

Reviewer #2: Yes

Reviewer #3: Yes

Reviewer #4: Yes

Reviewer #5: Yes

**********

Reviewer #1: Stigmatizing attitudes and knowledge of HIV/AIDS in the United Arab Emirates

Title-Incomplete, as the study population is not provided

Abstract-Well written

Methodology – remove the full form of UAE from the study design, as it is already stated, and use only abbreviations

The social media platform was utilised as a channel for recruiting participants. How did the authors ensure that the participants were UAE residents? State it in the methodology

Clearly specify the inclusion and exclusion criteria

The terms Awareness and knowledge were used interchangeably. Please use the term knowledge throughout the manuscript so that it reflects the title.

Rationalise the reason for piloting on 20 people

Specify how the level of knowledge was scored into good or poor, adequate or inadequate level of knowledge

Same with the stigmatization attitude, how the scoring was done, and how positive and negative attitudes were ascertained.

Remarks: Minor corrections

Reviewer #2: COMMENTS TO THE AUTHORS

Thank you for the opportunity to review this manuscript. The topic is important and relevant to HIV prevention, testing, disclosure, care-seeking and stigma reduction in the UAE and wider MENA region.

1. Title and scope of claims

The title is clear, but the manuscript should avoid implying national representativeness because the study used convenience sampling through social media. Please use cautious wording such as “among surveyed UAE residents” or “among study participants in the UAE” where appropriate.

2. Abstract

Please revise causal wording such as “driven by cultural and moral perspectives.” The study can identify associations, not causal drivers. Please also clarify that higher stigma scores indicate less stigmatizing attitudes.

3. Introduction

The introduction explains the importance of HIV stigma in the MENA region, but it should engage more directly with existing UAE-specific evidence, especially the previous UAE PLOS ONE study cited in the references. Please explain what this study adds beyond earlier UAE work.

Please strengthen the transition from regional background to the UAE context. Where available, include UAE-specific evidence on HIV epidemiology, testing access, stigma, disclosure, and the social or policy context.

4. Sampling and recruitment

Please describe the recruitment process more fully. State who distributed the survey, whether recruitment used personal, university, professional or community networks, whether paid promotion was used, how eligibility was assessed and how duplicate responses were prevented.

5. Generalisability

The sample is large but not representative of the UAE population. Young adults, students, females and residents of Sharjah and the northern emirates appear overrepresented. Please make this clear in the methods, discussion and limitations.

6. Sample size calculation

The Cochran formula can be presented as a target sample size estimate, but not as evidence of representativeness because the study did not use probability sampling. Please clarify this.

7. Consent and ethics

There is inconsistency between implied consent in the methods and written consent in the ethics statement. Please clarify whether consent was written, electronic or implied through questionnaire completion.

8. Questionnaire adaptation

Please explain how the questionnaire and SAT-PLWHA-S were adapted for the UAE context. State which items were modified, if any, and why.

9. Translation

Please provide more detail on the forward and backward translation process, including who translated the questionnaire, how discrepancies were resolved and whether conceptual equivalence was assessed.

10. Pilot testing

Please briefly describe the pilot participants, key feedback received and any revisions made after pilot testing.

11. HIV knowledge scoring

Please explain exactly how the HIV transmission knowledge score was calculated, since correct answers differ by scenario. A supplementary scoring table would be helpful.

12. Risk classification

Please review the “correct” risk categories for transmission scenarios. For example, “sex with condom” depends on correct and consistent use, while sharing a glass or being coughed on are not ordinary HIV transmission routes. Please align the wording and interpretation with accepted HIV transmission evidence.

13. HIV and AIDS terminology

The manuscript uses HIV, AIDS and HIV/AIDS in different places. Please standardise terminology and clarify why many questionnaire items refer to AIDS. This may influence stigma responses because AIDS may evoke more fear than HIV.

14. Stigma scale scoring

Please state consistently that higher stigma scores indicate less stigma. This should appear in the methods, results, table captions and figure captions.

15. Reliability and psychometrics

Please report internal consistency for the total stigma scale and subscales. If reliability differs between Arabic and English versions, this should also be reported.

16. Factor analysis

The discussion mentions factor analysis and a five-factor empirical solution, but this is not described in the methods or results. Please either report the factor analysis properly or remove this claim.

17. Missing data

Please explain why demographic analyses include 1,211 participants while knowledge and stigma analyses include 1,171. Clarify whether this was due to missing data, lack of HIV awareness or incomplete responses.

18. Statistical analysis

Please report whether assumptions for t-tests, ANOVA, correlation and linear regression were assessed. For the regression model, please also report checks for multicollinearity, especially between nationality and survey language.

19. Regression interpretation

Please avoid comparing the strength of predictors using unstandardised coefficients measured on different scales. If discussing predictor strength, consider reporting standardised coefficients.

20. Coding and reverse scoring

Please recheck reverse coding and the direction of subscale scores. The reported negative correlation between HIV knowledge and avoidance of personal contact appears counterintuitive if higher scores mean less stigma.

21. Results presentation

The results are informative but could follow the study objectives more clearly. Consider organising them under participant characteristics, HIV awareness and knowledge, stigma attitudes, knowledge-stigma relationship and predictors of stigma.

22. Tables and figures

Table 3 is very large and may be better placed in supplementary material. Table 4 contains a typo in “Oridnary.” Figure captions should clearly state that higher stigma scores mean lower stigma. The graphical abstract should also be checked for consistency.

23. Discussion

The discussion is relevant, but some interpretations are too strong for a cross-sectional convenience sample. Please avoid causal language and interpret findings in light of sampling limitations, possible social desirability bias and the demographic profile of participants.

24. Comparison with UAE evidence

Please compare the findings more directly with previous UAE evidence. Discuss whether similarities or differences may be due to sampling, timing, measurement tools, demographic composition or changes in public awareness.

25. Interpretation of knowledge and stigma

The association between higher HIV knowledge and lower stigma is important, but knowledge explained only part of the variation. Please emphasise that stigma may also be shaped by moral beliefs, fear, legal concerns, social norms and limited contact with people living with HIV.

26. Cultural interpretation

Please avoid broad statements about culture or religion. Interpret the findings respectfully and acknowledge diversity among UAE nationals, Arab expatriates and non-Arab residents.

27. Criminalisation

The findings on support for criminalisation are important, but the study measured attitudes rather than legal knowledge or detailed policy preferences. Please frame policy implications cautiously.

28. Contact with people living with HIV

The finding that knowing someone with HIV was associated with less stigma is useful. However, any discussion of contact-based interventions should acknowledge the need to protect confidentiality, safety and dignity of people living with HIV.

29. Healthcare professionals and students

The finding that healthcare professionals and health sciences students showed only marginally lower stigma deserves more discussion. This may suggest the need for stigma reduction, ethics and patient-rights content in health professional education.

30. Limitations

Please strengthen the limitations section. Explicitly mention non-representative sampling, self-selection bias, social desirability bias, measurement bias from scale adaptation and absence of data on HIV testing history, antiretroviral therapy knowledge, sexual orientation, migration status and prior HIV education.

31. Data availability

Please ensure that the dataset and codebook are included in the supporting information, in line with PLOS ONE requirements. If only aggregate data are provided, revise the data availability statement.

32. Language and style

The manuscript is generally readable, but minor typographical and style issues remain. Please correct errors such as “Oridnary” and standardise terms such as HIV, AIDS, HIV/AIDS, PLWH and PLWHA.

33. Conclusion

The conclusion should be more cautious. Educational campaigns are supported by the findings, particularly those addressing transmission misconceptions and condom effectiveness. Recommendations on policy reform and contact-based strategies should be framed as possible future intervention directions.

Reviewer #3: The manuscript addresses an important and relevant public health issue and contributes evidence from a setting where data remain relatively limited. It is well contextualized within the existing literature, with a clear rationale and objectives. Also, the use of a large sample size and a validated HIV stigma assessment tool strengthens the study. The analytical approach is generally appropriate, clearly described, and sufficiently detailed to support reproducibility. The manuscript is well organized, transparently reports its findings, and presents a thoughtful limitations section that acknowledges key study constraints, including those related to convenience sampling and potential selection bias.

While the study appears generally well conducted, several aspects of the manuscript would require some revision to enhance methodological transparency, reporting consistency, and overall clarity. Particularly, attention is needed regarding the reporting of study procedures, consistency across manuscript sections, description of analytical decisions, and a few formatting issues. Addressing the comments below would strengthen the rigor and interpretability of the manuscript.

Major comments

1. Consent procedures and ethics reporting: Please clarify the consent procedure. The Methods section indicates that participation constituted implied consent, whereas the Ethics Declaration states that written informed consent was obtained. These descriptions should be consistent throughout the manuscript. Additionally, the ethics statement appears incomplete and could be revised for clarity.

2. The manuscript reports 1,211 participants, but most of the HIV knowledge and stigma analyses appear to use N = 1,171. Please explain the reduction from 1,211 to 1,171 participants and clarify the reason for the exclusion of approximately 40 participants. A brief participant flow or explanation would improve transparency. Additionally, after reporting the minimum sample size (N = 385), clearly state how many participants were finally included in the study and analyses.

3. The Discussion section mentions factor analysis findings, including KMO and Bartlett’s test statistics; however, these analyses are not described in the Methods section nor reported in the Results section. Consider reporting the factor analysis methodology and findings appropriately or removing these statements.

Minor Comments

1. Introduction:

-Cite the source of the estimate of 38.4 million people living with HIV worldwide.

-Line 4/5: “The MENA region has one of the lowest reported HIV prevalence…” Consider reporting the prevalence and citing the source.

-Update UNAIDS statistics using the most recent available data. The argument regarding progress toward global HIV targets would remain valid while improving the current content of the manuscript.

2. Statistical analysis:

-OLS regression was performed; however, there is no discussion of the regression assumptions (e.g., multicollinearity, normality of residuals, and homoscedasticity). Consider including a justification statement about why the model was appropriate.

-Please justify model selection and report assessment of regression assumptions.

3. Discussion:

-Look out for wording that suggests representativeness of the UAE population, given the convenience sampling technique used.

4. Tables:

-For all tables, consider placing table titles above the tables.

-Given that the sample size is consistent within Tables 1 and 3, consider reporting N as a footnote below the table rather than repeating denominators throughout.

-Given the category headings already indicate “% (n)”, values could be reported as x% (y) rather than x% (n = y/N).

5. Figures:

-Figures 1 and 2 appear duplicated in the submission files. Please check and remove any duplicates if applicable.

Overall, the study addresses an relevant topic, especially in the UAE context and appears scientifically sound. Clarifying the issues above would strengthen the transparency, consistency, and readability of the manuscript.

Reviewer #4: This manuscript addresses an important and underexplored public health topic by examining HIV/AIDS knowledge and stigmatizing attitudes among residents of the United Arab Emirates. The relatively large sample and the examination of multiple dimensions of HIV-related stigma are strengths of the study. The manuscript is generally well organized and readable, and the findings have the potential to contribute useful evidence from a setting in which data on HIV-related stigma remain limited. However, several important methodological, analytical, and reporting issues currently limit the reproducibility and interpretation of the findings. In particular, the construction of the HIV knowledge score requires clarification; the participant flow and analytical sample sizes are not sufficiently explained; the factor analysis described in the Discussion is not reported in the Methods or Results and is not reconciled with the subsequent use of seven theoretical stigma dimensions; and several aspects of the statistical analyses and their reporting require verification. In addition, some interpretations and recommendations extend beyond what can be supported by the cross-sectional design and the variables measured. I therefore recommend that the authors address the following major points.

Major comments:

1. Questionnaire development and construction of the HIV knowledge score

The construction and scoring of the HIV knowledge measure require substantially greater methodological detail to ensure reproducibility and appropriate interpretation. The manuscript states that participants rated the perceived risk of HIV transmission across seven scenarios using four response categories (very low, low, high, and very high), and Table 2 identifies one response category as correct for each scenario. However, it is unclear how these item-level responses were converted into the overall knowledge score reported on a 0–100 scale. Please provide the complete scoring algorithm, including the numerical coding assigned to each response, whether items were scored dichotomously as correct/incorrect or according to their distance from the designated correct response, how item scores were combined, and how the final score was transformed to the 0–100 scale. The manuscript should also explain the basis for defining the correct level of risk for each scenario and provide appropriate supporting references.

Relatedly, the manuscript reports the mean knowledge score as “75.5% (SD=19.3)” while also describing it as a score on a 0–100 scale. Please clarify whether this value represents a transformed scale score or the percentage of correct responses, as these are not necessarily equivalent. The interpretation of 75.5 as “moderately high” also requires either a prespecified or validated interpretive criterion; otherwise, the authors should report the score descriptively without assigning an unsupported qualitative category. These clarifications are particularly important because the knowledge score is a central variable in the correlation and regression analyses and underlies several conclusions and comparisons with previous studies.

2. Participant flow, questionnaire skip logic, and analytical sample sizes

The manuscript should clarify the participant flow and explain the use of different analytical sample sizes across the study. A total of 1,211 participants were included, whereas several knowledge and stigma analyses were conducted using 1,171 participants. Based on the questionnaire skip logic, participants who selected “Not at all” when asked how knowledgeable they considered themselves about HIV/AIDS appear to have been directed past the objective knowledge items. If the 40-participant reduction from 1,211 to 1,171 reflects this skip pattern, this would plausibly explain the analytical sample used for the objective knowledge score and for analyses in which that score was required, including the knowledge–stigma correlations and the multivariable regression. However, this participant flow is not explicitly described in the manuscript.

Importantly, the same explanation does not appear to account for the use of N=1,171 in the descriptive stigma analyses, because the questionnaire indicates that participants who skipped the objective knowledge section should have proceeded to the stigma section. Similarly, the item asking whether participants knew someone with AIDS is reported for all 1,211 participants, although its position in the questionnaire appears to require clarification in relation to the stated skip logic. Please provide a transparent account of the number of participants entering each section of the questionnaire, the reasons for any exclusions, and the sample size used for each analysis. A participant flow diagram or a concise description in the Methods and Results would be helpful. The manuscript should also state how missing data were handled and whether the regression analysis used complete-case analysis.

3. Factor analysis and dimensional structure of the HIV stigma scale

The dimensional structure of the HIV stigma scale requires substantial clarification. The Discussion states that an exploratory factor analysis yielded a five-factor solution, whereas the Methods do not describe a factor analysis and the Results do not report its procedures or findings. At the same time, the manuscript presents and interprets stigma scores according to seven theoretical dimensions in the correlation analyses and Figure 1. It is therefore unclear which dimensional structure was used for scoring and interpretation and how the empirically derived five-factor solution relates to the seven theoretical dimensions.

Please report the factor analysis in the Methods and Results, including the rationale for conducting it, the extraction method, factor-retention criteria, rotation method, adequacy diagnostics, item loadings, treatment of cross-loadings, variance explained, and any items removed or reassigned. The authors should also explain whether the analysis was exploratory or confirmatory and provide evidence supporting the retained five-factor solution.

Most importantly, the authors should reconcile the five-factor empirical solution with the subsequent use of seven theoretical dimensions. If the seven-dimensional structure was retained despite the factor-analytic findings, the rationale for doing so should be clearly stated and supported, and the implications for the validity and interpretation of the dimension-specific scores should be discussed. Conversely, if the five-factor solution is intended to represent the structure of the scale in this sample, the subsequent analyses, figures, and interpretation should be aligned with that structure. The manuscript should consistently distinguish between theoretically defined subscales and empirically supported factors.

4. Statistical analysis and reporting of the multivariable model

Several aspects of the multivariable analysis require clarification and verification. First, the manuscript should provide a clearer description of how the final regression model was specified. Please state whether predictors were selected a priori based on a conceptual framework or previous evidence, or according to results from univariable analyses or another data-driven procedure. The manuscript should also ensure consistency regarding the variables included in the model. In particular, the Discussion refers to the healthcare field as not being significant in the multivariable analysis, but this variable does not appear in Table 4. Please clarify whether healthcare field was included in the fitted model and, if so, report the corresponding estimate; otherwise, the Discussion should not describe it as a finding from the multivariable model.

Second, the overall model statistics reported in Table 4 should be verified. The table reports N=1,171, R²=27.5%, adjusted R²=26.8%, and F(10, 1,171) =39.97. The denominator degrees of freedom would not ordinarily equal the total analytical sample size in a multiple linear regression with 10 predictor degrees of freedom and an intercept. In addition, the reported F statistic does not appear to be readily consistent with the stated N, R², and number of predictor degrees of freedom. Please verify and correct the model degrees of freedom and overall F statistic and ensure that all model summary statistics correspond to the same fitted model.

Third, the Methods should describe how the assumptions of ordinary least squares regression were assessed, including linearity, homoscedasticity, normality of residuals, influential observations, and multicollinearity. This is particularly relevant because the outcome is a bounded mean stigma score and Figure 2 suggests some asymmetry in its observed distribution. The distribution of the outcome alone does not determine the validity of OLS; therefore, the relevant residual diagnostics should be reported or summarized. The manuscript should also clarify how categorical reference groups were selected and how missing data were handled in the model.

5. Scoring and interpretation of the HIV stigma measure

The scoring and interpretation of the stigma measure requires further clarification. The manuscript states that higher scores indicate less stigmatizing attitudes, but Table 3 includes both positively and negatively worded items. Please describe the complete scoring procedure, clearly identify which items were reverse-coded, explain how item responses were combined into the overall and dimension-specific scores, and state how missing item responses, if any, were handled. This information is necessary to reproduce the overall stigma score, the dimension-specific scores, and the subsequent correlation and regression analyses.

The authors should also verify the direction and interpretation of the reported correlations between HIV knowledge and the stigma dimensions. In particular, the negative correlation between knowledge and the “avoidance of personal contact” dimension appears counterintuitive given that higher stigma scores are described as indicating less stigma and higher knowledge is otherwise associated with less stigmatizing attitudes. This result may be valid, but it raises the possibility of inconsistent item coding, reverse-scoring, or interpretation of the subscale direction. Please verify the coding and calculation of this dimension and, if the result remains unchanged, provide a clear interpretation of what the negative association means given the scoring direction.

More generally, the manuscript should ensure that the direction of all stigma scores is described consistently. Because some individual items are phrased in opposite directions, percentages of agreement cannot always be interpreted in the same way across items. The Results and Discussion should distinguish clearly between agreement with a particular statement and the direction of the derived stigma score.

6. Consistency and accuracy of the Results, tables, and figures

Several inconsistencies between the text, tables, and figures should be carefully checked. I recommend that the authors conduct a complete numerical cross-check of all reported percentages, numerators, denominators, and model statistics.

For example, in Table 3, for the item “Knowing transmission makes them criminals,” the table reports 256 participants who agreed and 692 who strongly agreed, totaling 948/1,171, which corresponds to approximately 81.0%, not 79.6%. Similarly, the reported interpretation of discomfort with an HIV-positive roommate should be checked against the actual wording and response distribution of the item “I would NOT bother if roommate was infected”; disagreement or strong disagreement with this statement totals 66.8%, rather than approximately 52%.

The tables and figures also require several presentational clarifications. In Table 2, the mean knowledge value is reported as “75.5% (SD=19.3)” although it is described as a score on a 0–100 scale; this should be clarified and reported consistently. In Table 3, the footnote states that higher scores indicate less stigma, but the table itself presents raw response distributions for items with different wording directions. The footnote should therefore clarify the relationship between the displayed responses and the coding used to calculate scores.

Figure 1 presents seven stigma dimensions despite the five-factor solution described in the Discussion, and this should be reconciled as noted above. In addition, the radial axis extends from 0 to 5 even though the stigma scale is described as ranging from 1 to 4; the axis should reflect the actual possible range of the scores. Figure 2 displays a histogram together with a smoothed curve, but the curve is not identified or explained in the caption. The caption should also describe the figure as the distribution of the overall stigma score rather than the “average overall stigma score.”

7. Interpretation of findings and conclusions

Several interpretations should be moderated to remain consistent with the cross-sectional design and the variables measured. The manuscript appropriately acknowledges in the Limitations that causal relationships cannot be established; however, causal or mechanistic language is still used in parts of the Discussion and Conclusion. Associations between knowledge and stigma should not be interpreted as evidence that educational interventions will reduce stigma or that “education alone is insufficient,” because the study did not evaluate an educational intervention. A more direct interpretation would be that HIV knowledge was associated with stigma but accounted for only part of the observed variation in stigmatizing attitudes.

Similarly, the association between knowing someone with HIV/AIDS and lower stigma is consistent with, but does not confirm, contact theory or demonstrate a stigma-reducing effect of personal contact. The measure appears to capture only whether participants knew someone with AIDS and does not assess the nature, closeness, duration, or quality of that contact. The relatively small number of participants reporting such contact should also be considered when interpreting this finding.

Interpretations of differences by nationality and survey language also require caution. The manuscript frequently attributes these associations to cultural or religious factors, although religiosity, cultural values, conservatism, and related constructs were not directly measured. Survey language may also reflect linguistic, educational, or other differences and, unless equivalence between the Arabic and English versions of the instrument has been demonstrated, possible differences in measurement should be considered. These findings should therefore be presented as associations that may warrant further investigation rather than as evidence of specific cultural or religious mechanisms.

The conclusions and recommendations should also be more closely aligned with the evidence generated by the study. Recommendations regarding educational campaigns may reasonably follow from the observed knowledge gaps, but more specific proposals involving healthcare-provider training, contact-based interventions, influencers, mobile applications, and telemedicine were not directly evaluated. Such recommendations should either be clearly framed as implications informed by external evidence and appropriately referenced or be moderated. The Conclusion should also avoid generalizing the findings to all UAE residents given the convenience sampling strategy and should instead refer to the study participants or sample where appropriate.

8. Data availability and reproducibility

The Data Availability Statement indicates that “All relevant data are within the manuscript and its Supporting Information files.” However, the materials provided appear to contain summary results and questionnaire information rather than the participant-level data underlying the reported means, correlations, factor analysis, and multivariable regression. Please ensure that the Data Availability Statement accurately reflects the materials that are available and that the data underlying the findings are provided in accordance with the journal’s data policy, subject to any justified ethical or confidentiality restrictions.

If participant-level data cannot be made publicly available because of consent, privacy, or ethics requirements, the manuscript should clearly state the nature of the restriction and describe an appropriate mechanism for data access, where permitted. The availability of scoring algorithms and sufficient documentation to reproduce the knowledge and stigma variables would also substantially improve reproducibility.

Minor Comments:

1. Please use the terms HIV and AIDS consistently throughout the manuscript. For example, Table 1 asks whether participants “know anyone with AIDS,” whereas the regression model refers to “knows someone with HIV.” These terms are not interchangeable, and the variable should be described according to the wording used in the questionnaire.

2. The statement that this is the “first comprehensive assessment of HIV knowledge and stigma in the UAE” should be justified or revised. The manuscript itself cites previous UAE research addressing public attitudes, knowledge, and stigma toward HIV/AIDS. The authors should specify precisely what aspect of the present study is novel.

3. Please avoid describing the mean knowledge score of 75.5 as “moderately high” unless a prespecified or validated threshold supports this classification.

4. The term “moral decline” should be used cautiously. The questionnaire appears to assess views concerning sexual freedom and traditional sexual values, rather than directly measuring a construct of moral decline. Interpretations should remain close to the wording and constructs measured.

5. The manuscript should consistently distinguish findings observed among the study participants from statements about UAE residents or the UAE population more broadly, given the convenience sampling strategy.

6. In the Limitations, the statement that anonymity and survey distribution “helped ensure authentic and genuine responses” is too strong. Anonymity may have reduced social desirability bias or encouraged more candid responses, but it cannot ensure response authenticity.

7. Please consider expanding the Limitations to acknowledge uncertainties related to the construction and validation of the knowledge score, the dimensional structure of the stigma measure, potential equivalence between the Arabic and English questionnaire versions, residual confounding from unmeasured cultural or religious constructs, and the limited measurement of personal contact with people living with HIV/AIDS.

8. Please clarify whether “recall bias” is a major concern for this study, as most questionnaire items appear to assess current knowledge, attitudes, and sociodemographic characteristics. Social desirability and response bias appear more directly relevant.

9. Please review the manuscript for minor grammatical and stylistic issues. For example, the sentence stating that distributing the survey across the Emirates and ensuring anonymity “helped ensure authentic and genuine responses” requires both grammatical revision and more cautious wording.

10. Please ensure that all tables and figures are fully self-contained, with all abbreviations, score directions, response coding, denominators, and graphical elements clearly defined in the corresponding titles or footnotes.

Reviewer #5: Well-crafted and insightful paper!

Well written abstract – clear and concise.

Introduction

- Between 2001 and 2012, the region saw a 73% increase in newly diagnosed individuals, the highest rate of increase globally (Ballouz et al., 2020). Since 2010, new infections have increased by 10-21% in the region, making it one of the few areas worldwide experiencing this alarming trend (Sallam et al., 2022).

o This is clear – however it could be good to provide context in terms of how this compares to other regions numerically over this time period for a deeper argument

- Under-reporting is widespread, and obtaining exact statistics is difficult as HIV remains a controversial topic in many MENA countries (Karbasi et al., 2022)

o Perhaps clarify what kind of information/data is underreported – prevalence, status, etc.

- Access to HIV testing, treatment, and care remains well below the global average, with only 67% of people living with HIV knowing their status and only 50% having access to treatment (UNAIDS Factsheet, 2022).

o I believe there is an updated regional profile form 2024 with more up to date data that may be beneficial to review

Methods

- It would benefit the reader to understand the process of the convenience sampling in more depth – how were the contacts from social media platforms acquired, any conditions in place that were required to be included, etc.

o What was the age cohort sought?

- Were both study information and questionnaire share digitally? – on what platforms? Over what time period was the questionnaire shared? Was that also from January to March?

- Great description of the questionnaire

- What language was the questionnaire available in?

Results

- Nice description of the results

- The tables are well done – clear and well organized

- The four figures do not have titles or associated numbers, and are not mentioned in the text. It would be beneficial to include a reference to them in the text and title them accordingly.

Discussion

- In the MENA region specifically, access to HIV testing, treatment, and care remains well below the global average, with only 67% of people living with HIV knowing their status and only 50% having access to treatment in 2022 (UNAIDS, 2022).

o Update figures to reflect the 2024 data

- Stigma and discrimination are recognized as major obstacles to achieving these targets, as they deter individuals from seeking testing and care and contribute to social isolation of those living with HIV (Stangl et al., 2013)

o The systematic review reference is strong but are there any regional specific resentences as well?

- The finding that 60.5% of participants overestimated the risk of protected sex is particularly concerning, as this misconception may undermine confidence in condoms as an effective HIV prevention method

o Perhaps adding in context around condom use in the region and any associated social/cultural factors

**********

what does this mean?). If published, this will include your full peer review and any attached files.

If you choose “no”, your identity will remain anonymous but your review may still be made public.

**Do you want your identity to be public for this peer review?**  For information about this choice, including consent withdrawal, please see our Privacy Policy

Reviewer #1: No

Reviewer #2: No

Reviewer #3: **Yes:**  Danilia Nellie Takang Fombang

Reviewer #4: No

Reviewer #5: No

**********

[NOTE: If reviewer comments were submitted as an attachment file, they will be attached to this email and accessible via the submission site. Please log into your account, locate the manuscript record, and check for the action link "View Attachments". If this link does not appear, there are no attachment files.]

To ensure your figures meet our technical requirements, please review our figure guidelines: https://journals.plos.org/plosone/s/figures

You may also use PLOS’s free figure tool, NAAS, to help you prepare publication quality figures: https://journals.plos.org/plosone/s/figures#loc-tools-for-figure-preparation.

NAAS will assess whether your figures meet our technical requirements by comparing each figure against our figure specifications.

Attachment Submitted filename: PLOSONE-Reviewer comments HIVAIDS.docx

Attachment Submitted filename: REVIEWER_REPORT_PONE-D-26-06560.docx

# Author comment: Author response to Decision Letter 1

**DOI:** 10.1371/journal.pone.0362376.r002

3 Sep 2026

Please refer to attached Response to Reviewers document

Attachment Submitted filename: Reviewers (1).docx

# Peer review: Decision Letter 1

**DOI:** 10.1371/journal.pone.0362376.r003

**Authors:** Daniel Demant

8 Oct 2026

Dear Dr. Abu Gharbieh,

Please submit your revised manuscript by Nov 22 2026 11:59PM. If you will need more time than this to complete your revisions, please reply to this message or contact the journal office at plosone@plos.org. When you're ready to submit your revision, log on to https://www.editorialmanager.com/pone/ and select the 'Submissions Needing Revision' folder to locate your manuscript file.

- A letter that responds to each point raised by the academic editor and reviewer(s). You should upload this letter as a separate file labeled 'Response to Reviewers'.
- A marked-up copy of your manuscript that highlights changes made to the original version. You should upload this as a separate file labeled 'Revised Manuscript with Track Changes'.
- An unmarked version of your revised paper without tracked changes. You should upload this as a separate file labeled 'Manuscript'.

If you would like to make changes to your financial disclosure, please include your updated statement in your cover letter. Guidelines for resubmitting your figure files are available below the reviewer comments at the end of this letter.

If applicable, we recommend that you deposit your laboratory protocols in protocols.io to enhance the reproducibility of your results. Protocols.io assigns your protocol its own identifier (DOI) so that it can be cited independently in the future. For instructions see: https://journals.plos.org/plosone/s/submission-guidelines#loc-laboratory-protocols. Additionally, PLOS One offers an option for publishing peer-reviewed Lab Protocol articles, which describe protocols hosted on protocols.io. Read more information on sharing protocols at https://plos.org/protocols?utm_medium=editorial-email&utm_source=authorletters&utm_campaign=protocols.

As the corresponding author, your ORCID iD is verified in the submission system and will appear in the published article. PLOS supports the use of ORCID, and we encourage all coauthors to register for an ORCID iD and use it as well. Please encourage your coauthors to verify their ORCID iD within the submission system before final acceptance, as unverified ORCID iDs will not appear in the published article. *Only*  the individual author can complete the verification step; PLOS staff *cannot*  verify ORCID iDs on behalf of authors.

We look forward to receiving your revised manuscript.

Kind regards,

Daniel Demant, PhD, MPH (Epidemiology), BA (Applied SocSci)

Academic Editor

PLOS One

Journal Requirements:

If the reviewer comments include a recommendation to cite specific previously published works, please review and evaluate these publications to determine whether they are relevant and should be cited. There is no requirement to cite these works unless the editor has indicated otherwise.

Please review your reference list to ensure that it is complete and correct. If you have cited papers that have been retracted, please include the rationale for doing so in the manuscript text, or remove these references and replace them with relevant current references. Any changes to the reference list should be mentioned in the rebuttal letter that accompanies your revised manuscript. If you need to cite a retracted article, indicate the article’s retracted status in the References list and also include a citation and full reference for the retraction notice.

[Note: HTML markup is below. Please do not edit.]

Reviewers' comments:

Reviewer’s Responses to Questions

**Comments to the Author**

Reviewer #3: All comments have been addressed

Reviewer #4: (No Response)

Reviewer #5: (No Response)

**********

2. Is the manuscript technically sound, and do the data support the conclusions?

Reviewer #3: Yes

Reviewer #4: Yes

Reviewer #5: Yes

**********

3. Has the statistical analysis been performed appropriately and rigorously?

Reviewer #3: Yes

Reviewer #4: Yes

Reviewer #5: Yes

**********

4. Have the authors made all data underlying the findings in their manuscript fully available?

The PLOS Data policy

Reviewer #3: Yes

Reviewer #4: Yes

Reviewer #5: Yes

**********

5. Is the manuscript presented in an intelligible fashion and written in standard English?

Reviewer #3: Yes

Reviewer #4: Yes

Reviewer #5: Yes

**********

Reviewer #3: The manuscript has improved substantially, and most of my previous concerns regarding sample size reporting, denominator discrepancies, updated epidemiological information, discussion framing, and table formatting have been satisfactorily addressed. The clarification regarding regression assumptions and the explanation for the reduction from N=1211 to N=1171 have also been adequately addressed.

A minor inconsistency remains in the description of the consent process. The Methods section refers to electronic informed consent, whereas the Declarations section refers to written informed consent. While the authors may wish to harmonize this wording for clarity and consistency, both sections clearly indicate that informed consent was obtained from participants. In my view, this discrepancy does not raise concerns regarding the ethical conduct of the study and does not affect the scientific rigor, validity, or interpretation of the findings.

Overall, I am satisfied that the authors have adequately addressed the substantive issues raised in the previous review, and I believe the manuscript is suitable for publication.

Reviewer #4: Overall comment

The authors have addressed most of the previous reviewer comments and have substantially improved the clarity and reporting of the manuscript. Two points remain to be addressed (the statistical results in Table 4 and the description of informed consent), and I would encourage the authors to resolve these before the manuscript is considered ready for publication.

Major comment

1. Statistical reporting in Table 4:

I noticed a potential discrepancy in the degrees of freedom reported for the regression model. The analysis includes 1,171 participants and 10 predictors, yet Table 4 reports F(10, 1,171) = 39.97. Given n = 1,171 and k = 10, the residual degrees of freedom would be expected to be 1,160 rather than 1,171. I would ask the authors to verify the degrees of freedom, the F-statistic, and the corresponding R² against the original regression output and correct them if necessary.

Minor comment

1. Consent statement:

The description of informed consent appears inconsistent across the manuscript. The Methods section states that electronic informed consent was obtained, whereas the Ethics Statement refers to “informed written consent.” I would ask the authors to clarify which term accurately reflects the procedure and to use consistent terminology throughout the manuscript.

Reviewer #5: This is a well-written study that provides relevant insights into the region, particularly given the increase in the number of cases reported. The findings are valuable in helping to better understand the regional context, especially in light of the limited data available for this particular region. The added value of the study is clear, and it provides concrete recommendations that are grounded in the findings.

Abstract – well written and clear

Introduction – well written and comprehensive covering importance context and clarifying the value add of this study

- “misconceptions are prevalent (Mumtaz et al., 2020)” – insight on the types of misconceptions captured?

- “despite some declining trends in recent years (Mumtaz et al., 2020)” – any reasons available to explain this decline?

- “Stigma has profound implications for HIV prevention and treatment, acting as a barrier to testing, disclosure, and care-seeking behaviors.” – the mental health aspect at an individual level may also important to acknowledge, I think that’s missing in the introduction and the discussion

Methods – clear and presented with all necessary information

- “Minor wording and formatting revisions were made based on participant feedbackf.” – just a minor spelling error

- During the pilot, did participants have an opportunity to provide feedback on the survey questions, including suggesting additional questions or identifying any questions that could potentially be removed or revised?

- How were the pilot participants selected?

Results and Discussion

- The results are clearly described and well discussed in the context of the literature in the discussion.

- It’s very useful that the authors identify several actionable recommendations

- One aspect that may be worth considering, depending on its relevance to the discussion, is how the stigma findings relate to the concentration of HIV among key populations, as noted in the introduction. It could be useful to reflect on whether this concentration may also intersect with broader social and cultural perceptions or stigma associated with these populations, and how this might influence the findings on HIV-related stigma.

**********

what does this mean?). If published, this will include your full peer review and any attached files.

If you choose “no”, your identity will remain anonymous but your review may still be made public.

**Do you want your identity to be public for this peer review?**  For information about this choice, including consent withdrawal, please see our Privacy Policy

Reviewer #3: **Yes:**  Danilia Nellie Takang Fombang

Reviewer #4: No

Reviewer #5: No

**********

[NOTE: If reviewer comments were submitted as an attachment file, they will be attached to this email and accessible via the submission site. Please log into your account, locate the manuscript record, and check for the action link "View Attachments". If this link does not appear, there are no attachment files.]

To ensure your figures meet our technical requirements, please review our figure guidelines: https://journals.plos.org/plosone/s/figures

You may also use PLOS’s free figure tool, NAAS, to help you prepare publication quality figures: https://journals.plos.org/plosone/s/figures#loc-tools-for-figure-preparation.

NAAS will assess whether your figures meet our technical requirements by comparing each figure against our figure specifications.

# Author comment: Author response to Decision Letter 2

**DOI:** 10.1371/journal.pone.0362376.r004

21 Oct 2026

Dear Editorial Board,

We would like to thank you and the reviewers for taking the time to review our paper (Manuscript ID PONE-D-26-06560R1) titled “HIV/AIDS knowledge and stigmatizing attitudes among adult residents of the United Arab Emirates: A cross-sectional study”, which was submitted to PLOS One. We appreciate your expertise and constructive feedback and hope to address it in an effort to make the paper more comprehensive. Revisions to the manuscript and replies to reviewers’ comments are outlined below.

Reviewer #3: The manuscript has improved substantially, and most of my previous concerns regarding sample size reporting, denominator discrepancies, updated epidemiological information, discussion framing, and table formatting have been satisfactorily addressed. The clarification regarding regression assumptions and the explanation for the reduction from N=1211 to N=1171 have also been adequately addressed.

We thank the reviewer for their careful reassessment and positive evaluation of our revisions. We appreciate the acknowledgment that the major methodological and reporting concerns have been satisfactorily addressed.

A minor inconsistency remains in the description of the consent process. The Methods section refers to electronic informed consent, whereas the Declarations section refers to written informed consent. While the authors may wish to harmonize this wording for clarity and consistency, both sections clearly indicate that informed consent was obtained from participants. In my view, this discrepancy does not raise concerns regarding the ethical conduct of the study and does not affect the scientific rigor, validity, or interpretation of the findings.

We agree and have standardized the description as “electronic informed consent.” We added a corresponding ethics-approval and informed-consent statement under Declarations, consistent with the online consent procedure described in Methods.

Overall, I am satisfied that the authors have adequately addressed the substantive issues raised in the previous review, and I believe the manuscript is suitable for publication.

Reviewer #4: Overall comment

The authors have addressed most of the previous reviewer comments and have substantially improved the clarity and reporting of the manuscript. Two points remain to be addressed (the statistical results in Table 4 and the description of informed consent), and I would encourage the authors to resolve these before the manuscript is considered ready for publication.

Major comment

1. Statistical reporting in Table 4:

I noticed a potential discrepancy in the degrees of freedom reported for the regression model. The analysis includes 1,171 participants and 10 predictors, yet Table 4 reports F(10, 1,171) = 39.97. Given n = 1,171 and k = 10, the residual degrees of freedom would be expected to be 1,160 rather than 1,171. I would ask the authors to verify the degrees of freedom, the F-statistic, and the corresponding R² against the original regression output and correct them if necessary.

Thank you for your comment. We have fixed the degrees of freedom in the table. The python package used does not calculate the degrees of freedom and it has to be calculated manually. The remainder of results remain as is

Minor comment

1. Consent statement:

The description of informed consent appears inconsistent across the manuscript. The Methods section states that electronic informed consent was obtained, whereas the Ethics Statement refers to “informed written consent.” I would ask the authors to clarify which term accurately reflects the procedure and to use consistent terminology throughout the manuscript.

Thank you for highlighting this inconsistency. The questionnaire required participants to affirm agreement electronically before entering the survey. We have used “electronic informed consent” consistently in the revision and added that terminology to the Declarations section.

Reviewer #5: This is a well-written study that provides relevant insights into the region, particularly given the increase in the number of cases reported. The findings are valuable in helping to better understand the regional context, especially in light of the limited data available for this particular region. The added value of the study is clear, and it provides concrete recommendations that are grounded in the findings.

Abstract – well written and clear

Introduction – well written and comprehensive covering importance context and clarifying the value add of this study

- “misconceptions are prevalent (Mumtaz et al., 2020)” – insight on the types of misconceptions captured?

We agree that the description was overly general. We have clarified the types of gaps identified in the regional synthesis, specifically knowledge of transmission modes and perceived risk. The Results and Discussion also give study-specific examples involving casual contact and condom-protected sex

- “despite some declining trends in recent years (Mumtaz et al., 2020)” – any reasons available to explain this decline?

Thank you. The referenced synthesis establishes persistent negative attitudes but does not provide sufficient evidence here to attribute changes over time to a specific cause. To avoid an unsupported temporal claim or speculative explanation, we removed the ambiguous “declining trends” statement.

- “Stigma has profound implications for HIV prevention and treatment, acting as a barrier to testing, disclosure, and care-seeking behaviors.” – the mental health aspect at an individual level may also important to acknowledge, I think that’s missing in the introduction and the discussion

We thank the reviewer for this valuable suggestion. We agree that HIV-related stigma can have important psychological consequences for people living with HIV. However, the primary aim of our study was to assess HIV-related knowledge and stigmatizing attitudes among adult residents of the UAE, rather than the psychological experiences of people living with HIV. As mental health outcomes were not assessed in our study, we believe that a detailed discussion of these effects would extend beyond the scope of our research. The Discussion already acknowledges broader consequences of stigma, including reduced quality of life and social isolation. We therefore prefer to maintain the current focus of the manuscript while recognizing mental health as an important area for future research.

Methods – clear and presented with all necessary information

- “Minor wording and formatting revisions were made based on participant feedbackf.” – just a minor spelling error

Thank you for noting the typo. The sentence has been corrected and clarified.

- During the pilot, did participants have an opportunity to provide feedback on the survey questions, including suggesting additional questions or identifying any questions that could potentially be removed or revised?

We thank the reviewer for this comment. As described in the Methods section, both language versions of the questionnaire were pilot tested with 20 participants to assess clarity, cultural appropriateness, conceptual equivalence, questionnaire flow, and approximate completion time. Minor wording and formatting revisions were made based on participant feedback. We believe the current description adequately reflects the purpose and outcomes of the pilot testing.

- How were the pilot participants selected?

We thank the reviewer for this comment. The 20 pilot participants were randomly selected members of the public who voluntarily participated in the pilot testing. Their feedback was used to assess the clarity, wording, and overall usability of the questionnaire before its distribution.

Results and Discussion

- The results are clearly described and well discussed in the context of the literature in the discussion.

- It’s very useful that the authors identify several actionable recommendations

- One aspect that may be worth considering, depending on its relevance to the discussion, is how the stigma findings relate to the concentration of HIV among key populations, as noted in the introduction. It could be useful to reflect on whether this concentration may also intersect with broader social and cultural perceptions or stigma associated with these populations, and how this might influence the findings on HIV-related stigma.

We agree. We added a cautious interpretation of how HIV-related stigma may overlap with attitudes about sexual behavior, substance use, and key populations. The discussion uses the observed response distribution for one stigma item and clarifies that respondents’ identities, experiences, and intersections of stigma were not measured, so no subgroup-specific inference is made.

Attachment Submitted filename: Reply to Reviewers.docx

# Editor report: Decision Letter 2

**DOI:** 10.1371/journal.pone.0362376.r005

**Authors:** Daniel Demant

25 Oct 2026

HIV/AIDS knowledge and stigmatizing attitudes among adult residents of the United Arab Emirates: A cross-sectional study.

PONE-D-26-06560R2

Dear Dr. Abu Gharbieh,

We’re pleased to inform you that your manuscript has been judged scientifically suitable for publication and will be formally accepted for publication once it meets all outstanding technical requirements.

Within one week, you’ll receive an e-mail detailing the required amendments. When these have been addressed, you’ll receive a formal acceptance letter and your manuscript will be scheduled for publication.

An invoice will be generated when your article is formally accepted. Please note, if your institution has a publishing partnership with PLOS and your article meets the relevant criteria, all or part of your publication costs will be covered. Please make sure your user information is up-to-date by logging into Editorial Manager at Editorial Manager® and clicking the ‘Update My Information' link at the top of the page. For questions related to billing, please contact billing support.

If your institution or institutions have a press office, please notify them about your upcoming paper to help maximize its impact. If they’ll be preparing press materials, please inform our press team as soon as possible -- no later than 48 hours after receiving the formal acceptance. Your manuscript will remain under strict press embargo until 2 pm Eastern Time on the date of publication. For more information, please contact onepress@plos.org.

Kind regards,

Daniel Demant, PhD, MPH (Epidemiology), BA (Applied SocSci)

Academic Editor

PLOS One

Additional Editor Comments (optional):

Reviewers' comments:

# Editor report: Acceptance letter

**DOI:** 10.1371/journal.pone.0362376.r006

**Authors:** Daniel Demant

PONE-D-26-06560R2

PLOS One

Dear Dr. Abu Gharbieh,

I'm pleased to inform you that your manuscript has been deemed suitable for publication in PLOS One. Congratulations! Your manuscript is now being handed over to our production team.

At this stage, our production department will prepare your paper for publication. This includes ensuring the following:

* All references, tables, and figures are properly cited

* All relevant supporting information is included in the manuscript submission,

* There are no issues that prevent the paper from being properly typeset

You will receive further instructions from the production team, including instructions on how to review your proof when it is ready. Please keep in mind that we are working through a large volume of accepted articles, so please give us a few days to review your paper and let you know the next and final steps.

Lastly, if your institution or institutions have a press office, please let them know about your upcoming paper now to help maximize its impact. If they'll be preparing press materials, please inform our press team within the next 48 hours. Your manuscript will remain under strict press embargo until 2 pm Eastern Time on the date of publication. For more information, please contact onepress@plos.org.

You will receive an invoice from PLOS for your publication fee after your manuscript has reached the completed accept phase. If you receive an email requesting payment before acceptance or for any other service, this may be a phishing scheme. Learn how to identify phishing emails and protect your accounts at https://explore.plos.org/phishing.

If we can help with anything else, please email us at customercare@plos.org.

Thank you for submitting your work to PLOS One and supporting open access.

Kind regards,

PLOS One Editorial Office Staff

on behalf of

Associate Professor Daniel Demant

Academic Editor

PLOS One
